# IL-32–producing CD8^+^ memory T cells define immunoregulatory niches in human cutaneous leishmaniasis

**DOI:** 10.1172/JCI182040

**Published:** 2025-05-15

**Authors:** Nidhi S. Dey, Shoumit Dey, Naj Brown, Sujai Senarathne, Luiza Campos Reis, Ritika Sengupta, Jose A.L. Lindoso, Sally R. James, Lesley Gilbert, Dave Boucher, Mitali Chatterjee, Hiro Goto, Shalindra Ranasinghe, Paul M. Kaye

**Affiliations:** 1 York Biomedical Research Institute, Hull York Medical School, University of York, York, United Kingdom.; 2 Department of Parasitology, Faculty of Medical Sciences, University of Sri Jayewardenepura, Gangodawila, Nugegoda, Sri Lanka.; 3 Department of Preventive Medicine, Instituto de Medicina Tropical de São Paulo, Faculdade de Medicina, Universidade de São Paulo, São Paulo, Brazil.; 4 Department of Pharmacology, Institute of Postgraduate Medical Education and Research, Kolkata, India.; 5 Secretaria de Saúde do Estado de São Paulo, Instituto de Infectologia Emílio Ribas, São Paulo, Brazil.; 6 University of São Paulo, Faculty of Medicine, Department of Infectious and Parasitic Diseases, São Paulo, Brazil.; 7 Technology Facility, Department of Biology, University of York, York, United Kingdom.

**Keywords:** Dermatology, Immunology, Infectious disease, Cellular immune response, Molecular pathology, Parasitology

## Abstract

Human cutaneous leishmaniasis (CL) is characterized by chronic skin pathology. Experimental and clinical data suggest that immune checkpoints (ICs) play a crucial role in disease outcome, but the cellular and molecular niches that facilitate IC molecule expression during leishmaniasis are ill defined. In Sri Lankan patients with CL, indoleamine 2,3-dioxygenase 1 (IDO1) and programmed death–ligand 1 (PD-L1) were enriched in skin lesions, and reduced PD-L1 expression early after treatment initiation was predictive of a cure rate following antimonial therapy. Here, we used spatial cell interaction mapping to identify IL-32–expressing CD8^+^ memory T cells and Tregs as key components of the IDO1/PD-L1 niche in Sri Lankan patients with CL and in patients with distinct forms of dermal leishmaniasis in Brazil and India. Furthermore, the abundance of IL-32^+^ cells and IL-32^+^CD8^+^ T cells at treatment initiation was negatively correlated with the rate of cure in Sri Lankan patients. This study provides insights into the spatial mechanisms underpinning IC expression during CL and offers a strategy for identifying additional biomarkers of treatment response.

## Abstract 2

Spatial cell interaction mapping in patient skin biopsies has identified IL-32+ CD8+ T cells as key components of an immunoregulatory niche in human leishmanaisis.

## Graphical abstract

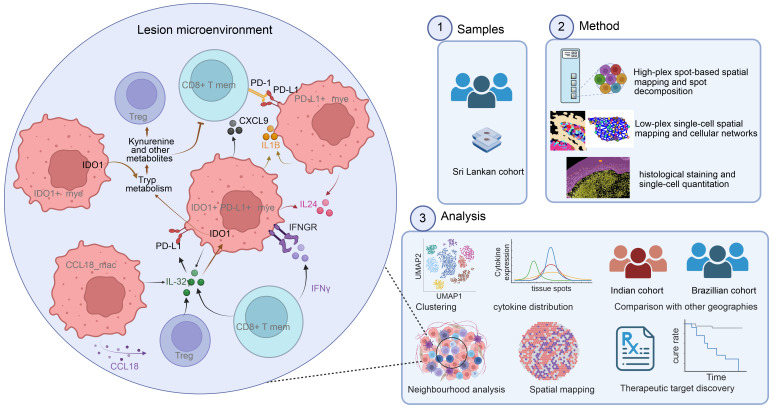

## Introduction

Cutaneous leishmaniasis (CL) is caused by protozoan parasites of the genus *Leishmania* and exhibits a wide spectrum of clinical presentations determined at least in part by the host immune response. *Leishmania* are intracellular parasites of myeloid cells — predominantly macrophages, monocytes, and DCs (1–3). Consequently, protective immunity is largely cell mediated, with effector CD4^+^ and CD8^+^ T cells producing cytokines (e.g., IFN-γ) that activate myeloid cell–intrinsic leishmanicidal activity (4, 5). Over-exuberant or persistent effector T cell responses at the site of infection promote tissue damage despite a reduction in parasite load (6). Conversely, T cell regulation and parasite-mediated subversion of macrophage epigenetic and transcriptional pathways (7) promote parasite persistence.

In leishmaniasis (8) and many other infectious (9) and noninfectious (10, 11) diseases, immune checkpoint (IC) pathways determine T cell effector function and disease outcome. Indoleamine-2,3-dioxygenase (IDO1) is a metabolic IC molecule expressed in response to inflammatory insults by macrophages (12), DCs (13) and B cells (14). IDO1 catalyzes the degradation of tryptophan to generate kynurenine and several other bioactive metabolites (15). Kynurenine acts through the aryl hydrocarbon receptor (AHR) to promote FoxP3-dependent Treg differentiation, while suppressing RORγt and Th17 development. Another key metabolite, kynurenic acid, suppresses Th17 responses, whereas 3-hydroxyanthranilic acid (3-HAA) enhances TGF-β signaling and Treg stability, while inhibiting Th1 and CD8^+^ T cell responses (15). In a murine model of CL, IDO1 attenuated local CD8^+^ and CD4^+^ T cell responses, whereas IDO1 ablation increased IL-6 and IL-17 production and decreased IL-10 production, correlating with reduced local inflammation and lower parasite burdens. Notably, IDO1 blocked IL-17 expression and prevented Tregs from converting into Th17-like T cells (16). Similarly, programmed death–ligand 1 (PD-L1) (encoded by *CD274*) is expressed by myeloid cells upon activation via LPS/IFN-γ or IL-4 and, when bound to PD-1 on T cells, inhibits activation and promotes IL-10 expression (17). These and other IC molecules have become invaluable prognostic and therapeutic targets in cancer (18, 19). In addition to modulating the natural course of disease, ICs may also play a role in tempering the efficacy of conventional but immune-dependent anti-infective and anticancer drugs. For example, we recently demonstrated that a decrease in PD-L1 expression early after treatment commencement was predictive of the rate of cure following treatment with sodium stibogluconate (SSG), a well-known immune-dependent, anti-leishmanial drug (20).

Despite many examples of disease-associated aberrant expression of IDO1 and PD-L1 (21–26), our understanding of the cellular and molecular pathways that regulate the expression of these IC molecules is largely derived or inferred from in vitro studies. Multiple cytokines and inflammatory signals can induce IDO1 (27, 28) and PD-L1 (29, 30) expression on human monocytes and DCs, including IFN-γ, TNF, TGF-β, IL-6, IL-10, IL-27, IL-32, pathogen-associated molecular patterns/damage-associated molecular patterns (PAMPs/DAMPs), and prostaglandin E2 (PGE2). IDO1 and PD-L1 are also induced by intracellular infection with *Leishmania* (20, 31, 32) and other pathogens (33, 34), suggesting that subversion of host cell function by manipulation of ICs is a conserved mechanism across pathogen evolution. There is currently little understanding, however, of how ICs are regulated in the complex spatial microenvironments associated with chronic infection or cancer.

Here, we combined multiple spatial methodologies to define cellular and transcriptomic niches containing IDO1^+^ and PD-L1^+^ myeloid cells in skin lesions from patients with CL with diverse forms of dermal leishmaniasis. We report diversity in niche composition and localization but identify CD8αβ^+^IL-32^+^ memory T cells and CD4^+^FOXP3^+^IL-32^+^ presumptive Tregs as common attributes of these immunoregulatory microenvironments.

## Results

### CD274 and IDO1 localize to myeloid cell–rich niches.

**Figure 1:**
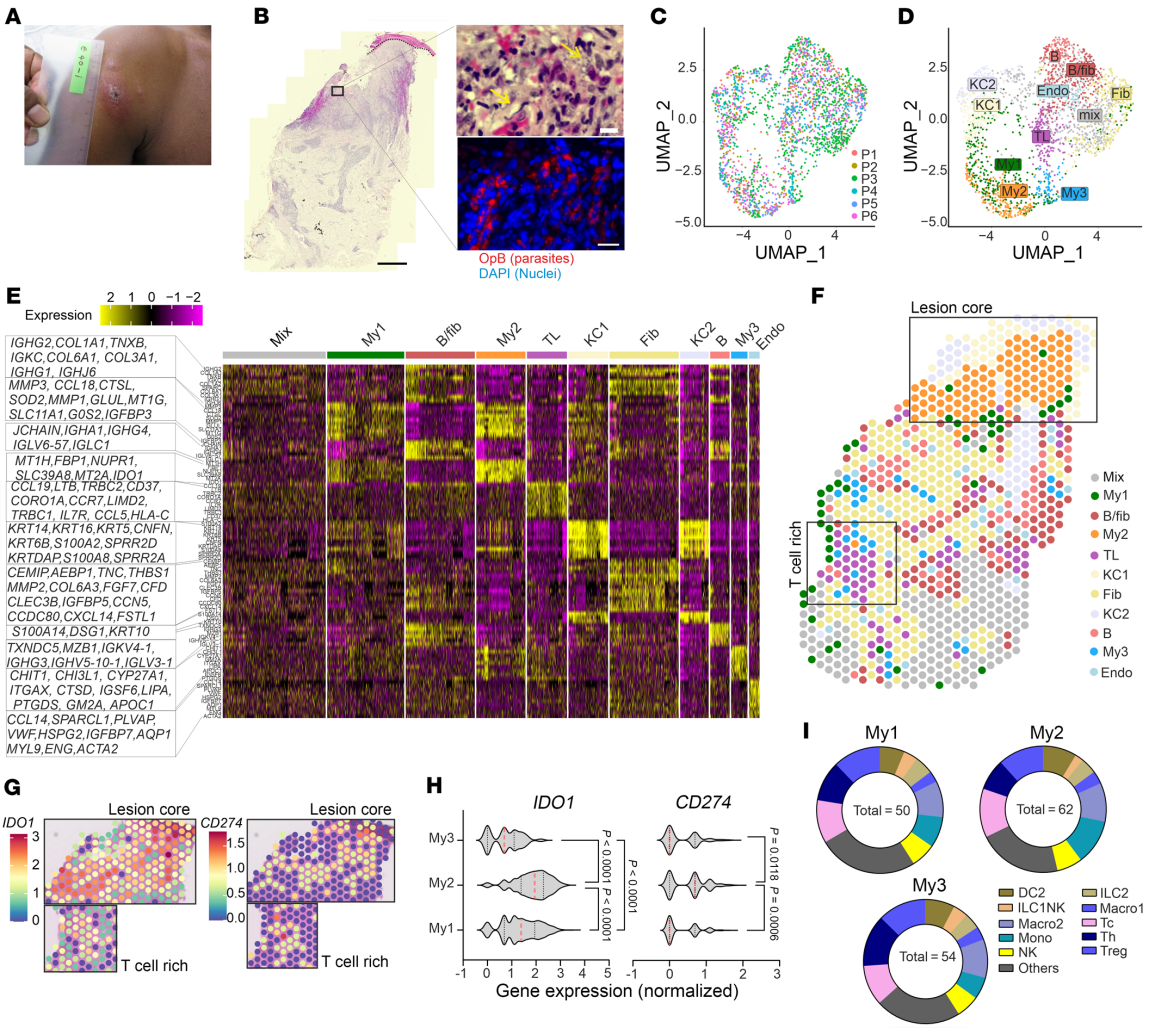
Visium analysis of spatial niches in *L. donovani* CL lesions. (**A** and **B**) P3 lesion. (**A**) Macroscopic lesion features. (**B**) Lesion histology. H&E insets on right show parasitism site (top, yellow arrows indicate parasites) and anti–1 OpB staining (bottom). Scale bars: 500 μm and 10 μm (insets). (**C** and **D**) UMAPs of Visium spots from SL CL P1–P6 colored by patient ID (**C**) and by gene expression (**D**). (**E**) Top 10 genes per cluster heatmap. (**F**) Spatial map for P3 showing clusters from **D**. (**G**) P3 spatial plots of normalized *CD274* (PD-L1) and *IDO1* gene expression. (**H**) *IDO1* and *CD274* violin plots for My1–3 clusters (red dotted lines indicate the mean; black dotted lines indicate the IC range; Kruskal-Wallis 1-way test). (**I**) Predicted average abundance for the 10 most abundant cell types in My1–3 spots with the average number of cells predicted as the total inside. Panels **C**–**E**, **H**, and **I** show data from 6 SL CL patients (P1–P6; Supplemental Table 1); data in other panels represent a single patient.

We conducted Visium spatial transcriptomics on formalin-fixed, paraffin-embedded (FFPE) sections from 6 patients (P1–P6) (Supplemental Table 1; supplemental material available online with this article; https://doi.org/10.1172/JCI182040DS1) (20), who presented with a papular and/or ulcerative plaque lesion typical of Sri Lankan CL (SL CL) (Figure 1A and Supplemental Figure 1A). Examination revealed dense cellular infiltration and parasitism in the papillary dermis (Figure 1B and Supplemental Figure 1B). A total of 2,418 Visium spots (median of 4,104 gene counts per spot; Supplemental Figure 1C) were colored by cluster identities and visualized in the uniform manifold approximation and projection (UMAP) space, with all patients represented in each cluster (Figure 1C). Eleven high-level clusters were annotated on the basis of transcript abundance: myeloid cells (My1, My2, My3), B cells/fibroblasts (B/fib), T cells (TL), keratinocytes (KC1 and KC2), fibroblasts (Fib), B cells (B), endothelial cells (Endo), and an uncharacterized cluster (mix) (Figure 1, D and E). We generated a coarse transcriptomics map reflecting underlying tissue morphology (Figure 1F and Supplemental Figure 1, D and E). The 3 myeloid-rich clusters had distinct positioning and gene expression signatures. My1 (*SELENOP*^+^) and My2 (*CCL18*^+^) localized to papillary dermis near the epidermal-dermal junction (herein referred to as the lesion core). My1 and My2 were enriched for mRNAs encoding (a) S100 proteins (*S100A8*, *S100A9*), suggestive of neutrophils, monocytes, and DCs (35); (b) metallothionein genes (*MT1H*, *MT1G*, *MT1X*, and *MT2A*, and *SLC39A8*), suggestive of altered metal ion homeostasis; and (c) *CCL18*, a T cell chemoattractant (36) (Supplemental Figure 2A). My1 also contained B cells (*IGHA1*, *IGHG2*, *IGKC*) (Supplemental Figure 2A). My2 was also enriched for other immune regulators including *SIGLEC10* (37), *VSIG4* (38), and *CD300E* (39) (Supplemental Figure 2A). My3 (*CHIT1*^+^) was associated with a T lymphocyte–rich cluster (*CCL19*^+^) deeper in the dermis (Figure 1F). *IDO1* and *CD274* transcripts mapped to the lesion core as well as to the T cell–rich region (Figure 1G) and the My1, My2, and My3 clusters (Figure 1H).

Using a publicly available scRNA-Seq dataset (26) and the Cell2Location (40) prediction tool, we estimated the abundances of 40 cell types (top 20 shown in Supplemental Figure 2B). My1, My2, and My3 clusters exhibited cellular heterogeneity, as they contained cytotoxic T (Tc) cells (*CD3D*, *CD8A*, *CD8B*), Th cells (*CD4*, *IL7R*, *CD40LG*, *PTGER4*), Tregs (*CD4*, *FOXP3*, *TIGIT*, *BATF*, *CTLA4*), NK cells (*KLRD1*, *GNLY*, *PRF1*, *GZMB*, *NKG7*), myeloid DCs (DC2; *CD68*, *NR4A1*, *NR4A2*, *CLEC10A*, *FCGR2A*, *CD83*^lo^), group 2 innate lymphoid cells (ILC2s) (*IL7R*, *PTGDR2*, *GATA3*), macrophages (Macro1) (*MARCO*, *CD163*, *C1QB*, *FCGR2A*), Macro2 (*FCGR2A*, *F13A1*, *NR4A1*, *NR4A2*, *KLF4*), monocytes (*CD14*, *IL1B*), and ILC1/NK cells (*KLRB1*, *XCL1*, *XCL2*, *TNFRSF18*, *TNFRSF11*, *FCER1G*, *KIT*), reflecting the diverse cell types present in these myeloid-rich niches (Figure 1I and Supplemental Figure 2, C–L). Although Ig transcripts were found in these clusters, formal identification of B cells and plasma cells was not possible using the Reynolds et al reference data set (26).

### Mature DCs have abundant CD274 and IDO1 mRNAs.

**Figure 2:**
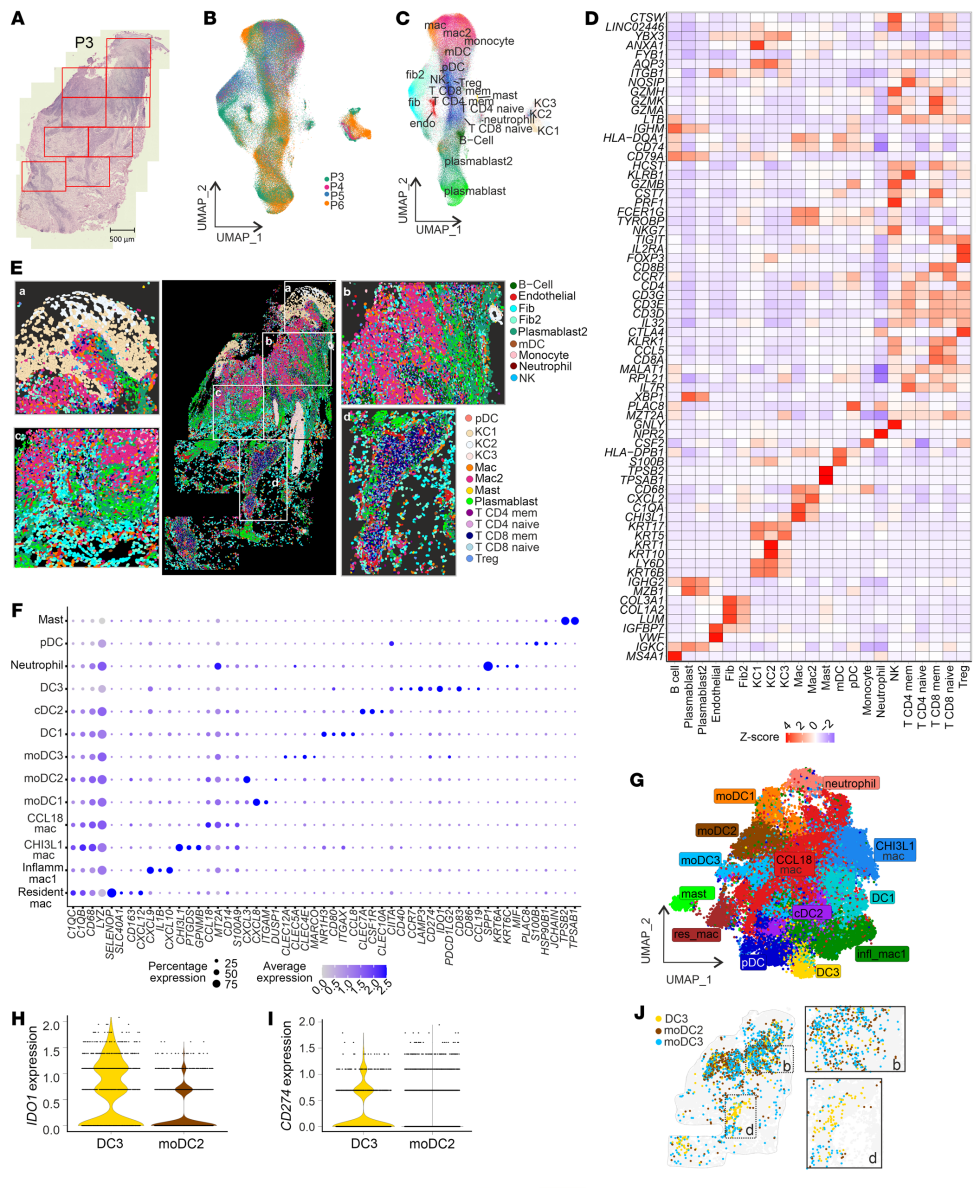
CosMx single-cell transcriptomics imaging of cutaneous lesions from Sri Lankan patients. (**A**–**D**) Single-cell spatial mapping of CL lesions. (**A**) H&E-stained image of a lesion from patient P3 showing FOVs analyzed using NanoString CosMx. Scale bar: 500 μm. (**B**–**D**) UMAP by patient number (**B**) and cell type (**C**), and cell-type–specific top genes heatmap (**D**). (**E**) Single-cell map of stitched FOVs of P3 biopsy with magnified regions (boxes a–d). mDC, myeloid DC; pDC, plasmacytoid DC; mem, memory. (**F**) Gene expression dot plot across myeloid subtypes. (**G**) UMAP of subclustered myeloid cells. res, resident; infl, inflammatory; cDC2, type 2 conventional DCs. (**H** and **I**) Violin plots showing *IDO1* (**H**) and *CD274* (**I**) expression in the top 2 highest-expressing cells, sorted by average cluster expression level. (**J**) Spatial localization of DC subset: DC3 and monocyte-derived DC subsets moDC2 and moDC3 in P3 with magnified regions. Panels **B**–**D** and **F**–**I** show data from 4 SL CL patients (P3–P6; Supplemental Table 1); other panels represent data from a single patient.

To overcome limitations of cellular deconvolution and to ascertain the spatial arrangement of cell types in Visium niches, we performed single-cell spatial transcriptomics analysis (NanoString CosMx SMI) (41). We analyzed 115,157 single cells (from 20 fields of view [FOVs]) derived from 4 patients (~46,000, 11,000, 34,000, and 39,000 cells from P3–P6, respectively) (Figure 2, A and B, Supplemental Figure 3, A and B, and Supplemental Table 1) and identified 22 cell clusters (Figure 2, C and D). Cells with myeloid signatures were most abundant in the papillary dermis (Figure 2E), supporting our Visium analysis. We also identified T cells near the dermal and epidermal boundary, flanked by fibroblasts (*COL1A1*, *COL6A2*, *COL6A1*, *COL3A1*, *LUM*) and B cells (*IGHG*, *IGKC*, *IGHM*, *CD79A*) in the lesion core. Deeper in the dermis, T cells, along with scattered macrophages, were dominant (Figure 2E). Localization of T cell (*CD3D*/*E*/*G*), NK (*NKG7*, *GNLY*, *GZMH*/*A*, *CCL5*) and Treg (*CD3D/G*^+^, *FOXP3*^+^, *CD4*^+^, *IL2RA*^+^, *TIGIT*^+^, *CTLA4*^+^) populations are shown in Supplemental Figure 3, B–D. We next subclustered all myeloid cells to obtain 13 subpopulations (Figure 2, F and G, and Supplemental Figure 3, B, E, and H). When ranked by *CD274* and *IDO1* mRNA abundance (Figure 2, H and I), the mature DC cluster DC3 (*CD80*, *LAMP3*, *CCR7*) was identified as having the highest proportion of *IDO1*^hi^*CD274*^hi^ cells. Other myeloid cell populations also expressed these IC molecules to a lesser extent. DC3 mapped to the lesion core and the deeper T cell–rich regions of the dermis (Figure 2J) and expressed mRNA for other immunoregulatory molecules (*CD40* and *PDCD1LG2*; PD-L2; Figure 2F).

### CD274 and IDO1 mRNA abundance in Visium spots correlates with distinct cytokines and chemokines.

**Figure 3:**
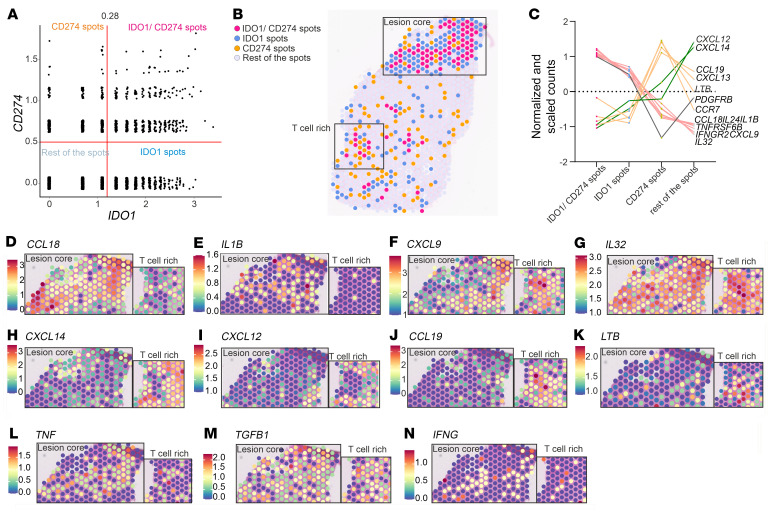
Visium 55 μm neighborhoods of *IDO1*^+^ and *CD274*^+^ spots. (**A**) *IDO1* and *CD274* normalized gene expression scatter plot for all Visium spots with thresholds (*x* = 1.1, *y* = 0.5) defining IDO1, CD274, IDO1/CD274, or other spot classes. (**B**) P3 spatial plot by spot class in **A**. Insets identify the lesion core and dermal T cell–rich regions as inferred from Visium and CosMx datasets. (**C**) Cytokine, chemokine, and receptor abundance by the expression classes described in **A**. (**D**–**K**) Spatial feature plots for cytokines, chemokines, and ILs from **C** in P3’s lesion core and T cell–rich area. (**L–N**) Additional selected gene spatial features in the same regions. Panels **A** and **C** show data from 6 SL CL patients (P1–P6; see also Supplemental Table 1); others represent data from a single patient.

To gain insights into the pathways leading to IDO1 and PD-L1 expression, we identified cytokine expression in niches where *CD274*^+^ and *IDO1*^+^ cells were abundant. Given the limited cytokine/chemokine coverage of the CosMx gene panel, we reverted for this analysis to our Visium data. We separated the Visium spots into 4 classes: *CD274*^hi^*IDO1*^lo^ (CD274 spots), *CD274*^lo^*IDO1*^hi^ (IDO1 spots), *CD274*^hi^*IDO1*^hi^ (IDO1 and CD274 spots), and *CD274*^lo^*IDO1*^lo^ (rest of the spots) and mapped their distributions and predicted cell type abundances (Figure 3, A and B). We performed differential (*P* < 0.05) expression analysis to identify cytokines, chemokines, their receptors, and IC mRNAs that were significantly different across these classes (Supplemental Figure 4A) and observed 4 distinct patterns of gene expression (Figure 3, C–K). *CCL18*, *IL24*, *IL1B*, *TNFRSF6B*, *IFNGR2*, *CXCL9*, and *IL32* mRNAs were abundant in IDO1/CD274 spots and to a lesser degree in IDO1 spots in the lesion core, with *CXCL9* and *IL32* mRNAs also found in the T cell–rich hypodermis (Figure 3, D–G). mRNAs for the homeostatic chemokines *CXCL12* (42) and *CXCL14* (43) had a reciprocal distribution (Figure 3, H and I). *CCL19* (Figure 3J), *CCR7*, *CXCL13*, and *LTB* (Figure 3K) were most abundant in CD274 spots in T cell–rich areas. Cell deconvolution analysis revealed that Tc cells, Tregs, Th cells, monocytes, and Macro2 cells were enriched in IDO1, IDO1/CD274, and CD274 spots (Supplemental Figure 4B). Amont anti-leishmanial effector and regulatory cytokines (1), *TNF* and *IFNG* were concentrated in the lesion core while *TGFB1* was widespread across the tissue (Figure 3, L–N).

### CD8^+^ T memory cells and Tregs are neighbors of CD274- and IDO1-expressing cells.

**Figure 4:**
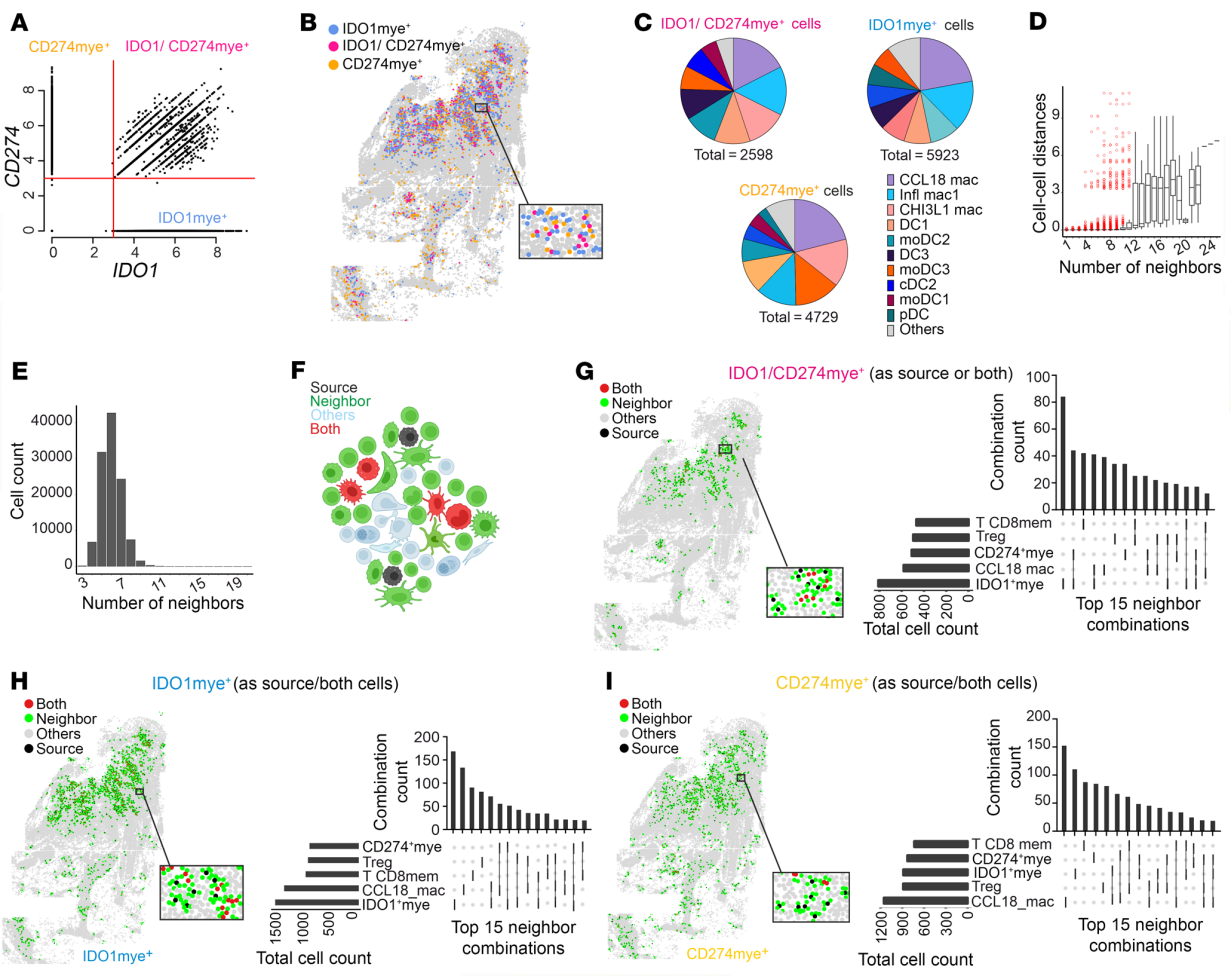
CosMx single-cell analysis of *IDO1*^+^ and *CD274*^+^ myeloid cell phenotypes and neighboring cells. (**A**) Myeloid cell *CD274* and *IDO1* expression scatter plot from the CosMx dataset (thresholds: *x*,*y* = 3). (**B**) P3 Spatial plot by classes from **A**. (**C**) Myeloid subset distribution in IDO1^+^CD274mye^+^, IDO1mye^+^, and CD274mye^+^ cells. (**D** and **E**) Cell distances (IQR + median) (**D**) and neighbor count (range, 3–22) (**E**) in the Delaunay network analysis. (**F**) Cartoon representation of neighborhood analysis (see Methods for details). (**G**–**I**) Spatial maps (left) and UpsetR plots (right) showing neighbor interactions for IDO1mye^+^CD274mye^+^ (**G**; *n* = 2,418 pairs), IDO1mye^+^ (**H**; *n* = 5,370), and CD274mye^+^ (**I**; *n* = 4,308) (see also the Supporting Data Values file). UpsetR plots show the top 15 heterotypic interactions; connecting lines indicate combinations, with vertical bars showing combination totals and horizontal bars showing total neighbor counts per cell type. For panels **A**, **C**–**E**, and **G**–**I**, the right panels show data from 4 SL CL patients (P3–P6; Supplemental Table 1); other panels represent data from a single patient.

To identify cellular interactions contributing to these niches, we similarly separated myeloid cells in our CosMx dataset into 4 classes (Figure 4, A and B). CCL18^+^ macrophages (*CCL18*, *MT2A*, *CD14*, *S100A9*, *C1QB*, *CD68*, lysozyme [*LYZ*]) were the predominant cell type across all 3 classes (Figure 4C). We then used Delaunay triangulation in Giotto to construct a pan-spatial network based on cell centroid physical distances (44). We observed that each cell had 4–8 close “neighbors” (Figure 4, D and E). Using this framework, we assigned IDO1^+^mye, CD274^+^mye, and IDO1^+^CD274^+^mye cells as “source” cells or as “both” neighbor and source (Figure 4F) and visualized source-neighbor or both-neighbor abundance using the UpSetR tool (45) (Figure 4, G–I). This analysis suggested a consistent neighborhood composition, comprising other IDO1^+^ or CD274^+^ myeloid cells, CD274^–^IDO1^–^CCL18^+^ macrophages, CD8^+^ memory T cells (defined by *CD3D/E^+^*, *CD8A/B*^+^, *CST7^+^*, *NKG7^+^*, *GZMH^+^*, *GZMA*^+^, *GZMK*^+^, *CCL5*^+^ expression), and Tregs (defined by *CD3D/G*^+^, *FOXP3^+^*, *CD4^+^*, *IL2RA*^+^, *TIGIT^+^*, *CTLA4^+^* expression and an absence of *CD8* expression) (Figure 4, G–I). These CD8^+^ T memory and Tregs also expressed *LAG3* (21.7% and 39.2%, respectively), *TIGIT* (12.9% and 13.3%, respectively), and *PDCD1* (PD-1; 12.3% and 12.7%, respectively) suggesting a potential inhibitory microenvironment surrounding IDO1^+^ and/or CD274^+^ myeloid cells (Supplemental Figure 5A).

### Validation of cellular interactions between IDO1, CD274 myeloid cells, and neighbors.

**Figure 5:**
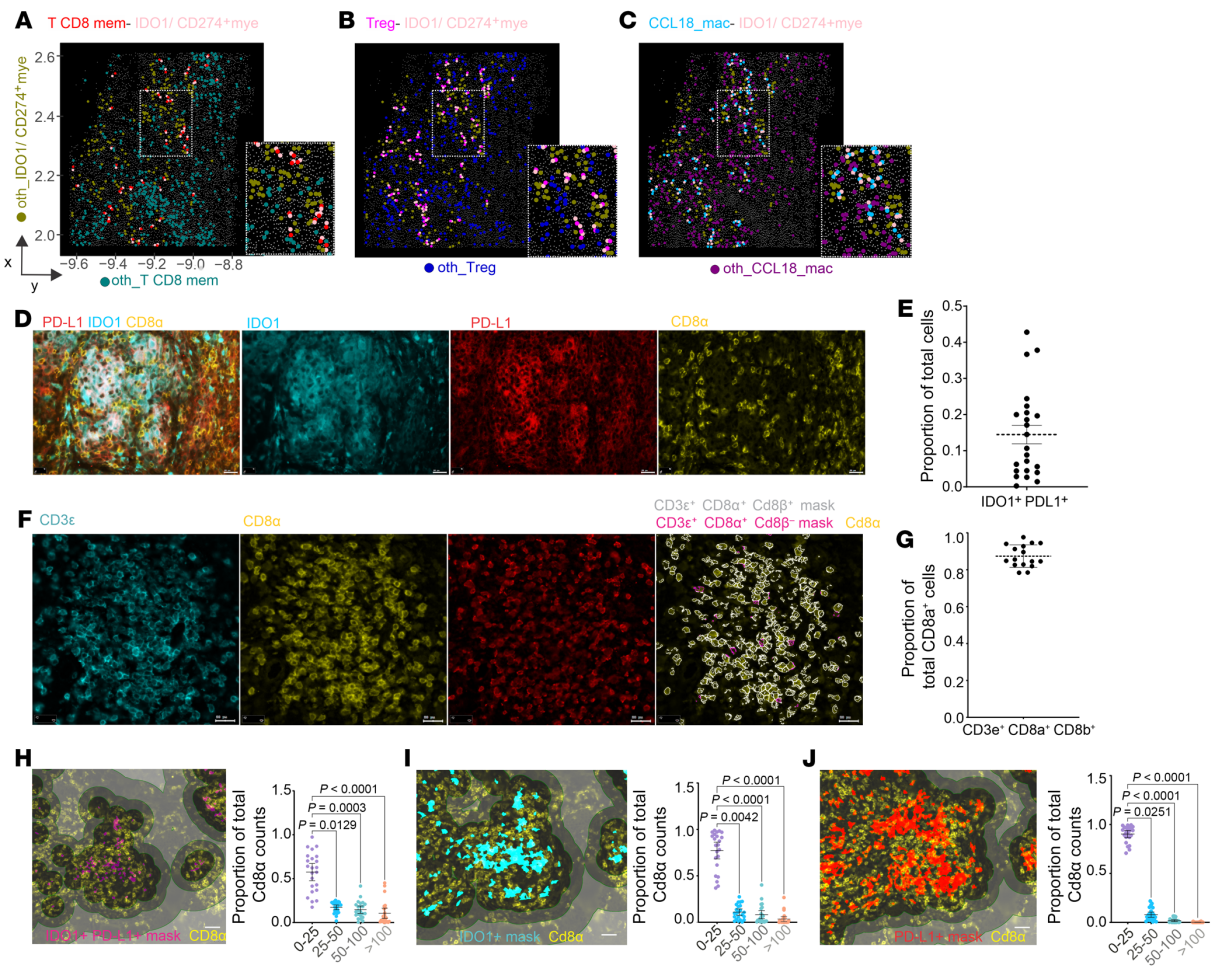
CosMx and IHC validation of cellular neighborhoods. (**A**–**C**) Representative FOVs from the CosMx transcriptomics dataset showing IDO1mye^+^CD274^+^mye cells (pink) interacting with CD8^+^ memory T cells (T CD8 mem; red) (**A**), Tregs (magenta) (**B**), and CCL18 macrophages (CCL18_mac; blue) (**C**). Noninteracting cells are shown with an “oth_” prefix. (**D**) IHC images showing IDO1, PD-L1, and CD8a protein expression. Scale bars: 20 μm. (**E**) Proportion of cells coexpressing IDO1 and PD-L1 from **D**. (**F**) IHC images showing CD3ɛ, CD8α, and CD8β protein expression. Scale bars: 20 μm. (**G**) CD3ɛ^+^CD8α^+^CD8β^+^ T cells/mm² as a proportion of total CD8α^+^ cells (*n* = 19). (**H**–**J**) CD8α proximity to IDO1^+^PD-L1^+^ (**H**, magenta), IDO1^+^ (**I**, cyan), and PD-L1^+^ cells (**J**, red). Images in **H**–**J** show distance masks in shades of gray at 25, 50, and 100 μm diameter. Graphs in **H**–**J** show the proportions of CD8^+^ T cells by distance (Friedman’s test with Dunn’s adjustment, mean ± SD). Scale bars: 20 μm (**D** and **F**) and 40 um (**H**–**J**). For panels **E** and **H**–**J**, the right panels show data from 23 SL CL patients (see also Supplemental Table 1); other panels represent data from a single patient.

To validate these findings, we first spatially mapped different combinations of neighboring cell types onto the tissue, offering a visual representation of other IDO1^+^ or CD274^+^ myeloid cells, CD274^–^IDO1^–^CCL18^+^ macrophages, CD8^+^ memory T cells, and Treg interactions within the tissue microenvironment (TME) (Figure 5, A–C). For orthogonal validation, we performed immunostaining for PD-L1, IDO1, and CD8α proteins in biopsies in which sufficient tissue was available (*n* = 23 SL CL patients; Supplemental Table 1). CD8α^+^, IDO1^+^, PD-L1^+^, and IDO1^+^PD-L1^+^ cells were spatially colocated (Figure 5, D and E). We confirmed that the majority of CD8α^+^ T cells also expressed CD3ɛ and CD8β (87.4% ± 0.6%; mean ± SD; Figure 5, F and G). Applying bespoke image analysis pipelines, we created image masks at distances of 25, 50, and 100 μm from IDO1^+^PD-L1^+^, IDO1^+^, and PD-L1^+^ cells (Figure 5, H–J), which revealed that the majority of CD8^+^ T cells were located within 25 μm of such cells and confirming them to be immediate neighbors.

### IL-32–expressing CD8^+^ memory T cells and Tregs colocalize with IDO1- and CD274-expressing myeloid cells.

**Figure 6:**
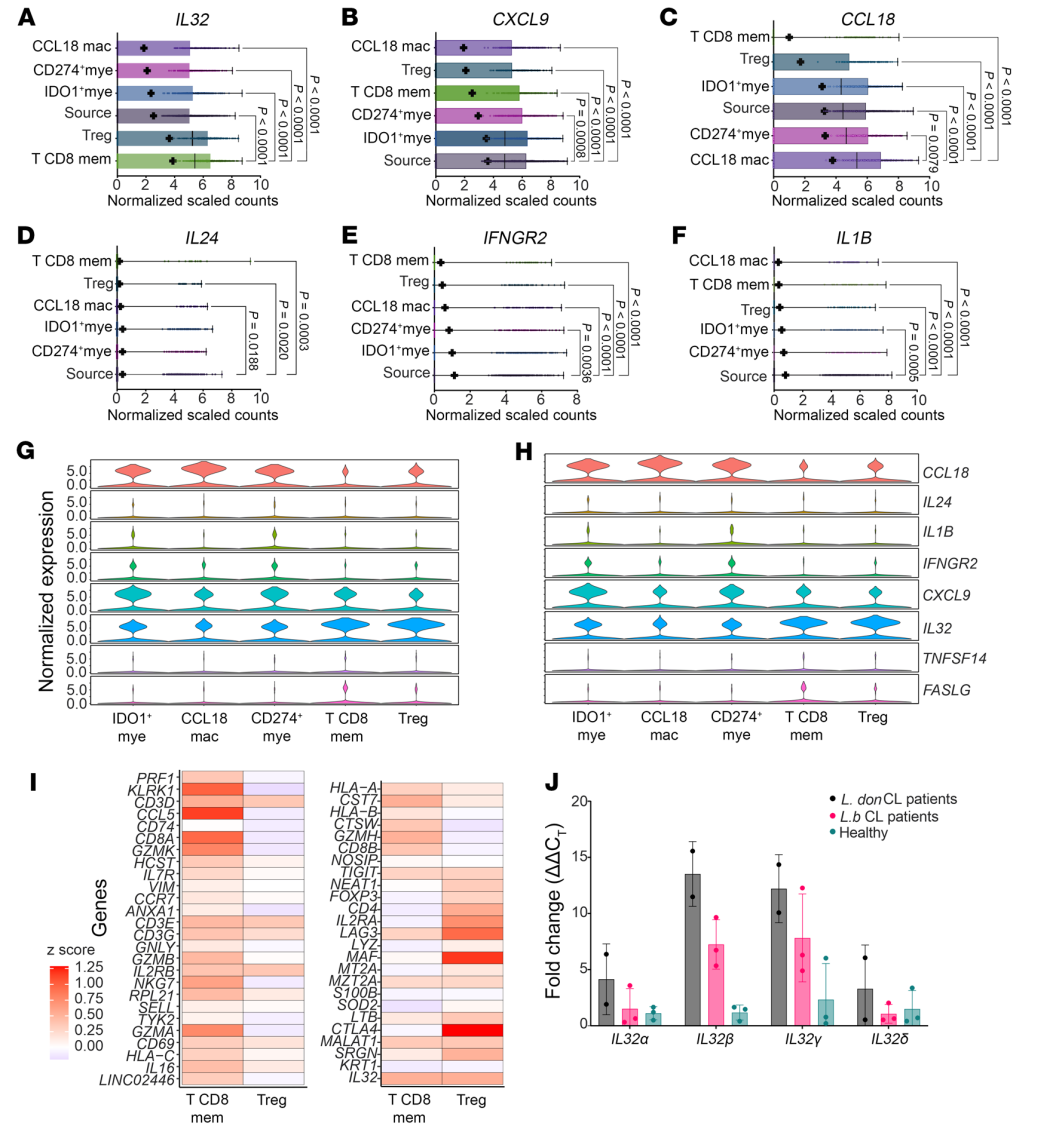
CosMx analysis of cellular sources and targets of cytokines in IDO1^+^ and CD274^+^ microenvironments. (**A**–**F**) Expression of *IL32* (**A**), *CXCL9* (**B**), *CCL18* (**C**), *IL24* (**D**), *IFNGR2* (**E**) and *IL1B* (**F**) in the top 5 neighbors from Figure 4G. Data are presented as follows: mean = +, median = vertical line; Kruskal-Wallis with Dunn’s correction. (**G** and **H**) Expression of *CCL18*, *IL24*, *IL1B*, *IFNGR2*, *CXCL9*, *IL32*, *TNFSF14*, and *FASLG* in the top 5 neighbors of IDO1mye^+^ and CD274mye^+^ cells. (**I**) Heatmap showing the phenotype of neighboring CD8^+^ memory T cells and Tregs. Scale shows Gini coefficient *z* scores. Data shown in **A**–**I** are from 4 SL CL patients (see also Supplemental Table 1). (**J**) *IL32* isoform (α, β, γ, and δ) fold change versus *GAPDH* and healthy skin (*n* = 3) in *L. donovani* (*L. don*) CL patients (*n* = 2) and *L*. (*V.*) *braziliensis* (*L. b*) CL patients (*n* = 3). Data indicate the mean ± SD.

We then examined the phenotypes of source and neighboring cells with respect to the cytokines and chemokines identified above as coenriched in *CD274* and *IDO1* niches (Figure 3C). *IL32* mRNA abundance was higher in CD8^+^ T memory cells and Tregs, whereas *CXCL9* and *CCL18* mRNAs were abundant in other myeloid cells (Figure 6, A–C). In contrast, *IL24*, *IFNGR2*, and *IL1B* mRNAs were primarily found in *IDO1*^+^*CD274*^+^ and source myeloid cells (Figure 6, D–F). Similar patterns were observed for neighbors of *CD274* and *IDO1* single-positive cells (Figure 6, G and H). Hence, in SL CL lesions, CD8^+^ memory T cells and Tregs (Figure 6I) represented the most common neighbors of *CD274*^+^*IDO1*^+^ cells and were a major source of the *CD274*- and *IDO1*-inducing cytokine IL-32. Since both *IL32B* and *IL32G* isoforms can induce either IDO1 or/and PD-L1 in macrophages (46–48) and secretory IL-32γ expression has been reported in patients with *Leishmania* (*Viannia*) *braziliensis* infection (49), we investigated which isoform was expressed in SL CL lesions. Quantitative real-time PCR (qRT-PCR) confirmed that *IL32G* and *IL32B* were the most highly upregulated isoforms in lesion compared with healthy skin (Figure 6J and Supplemental Tables 1 and 2).

### IL32 is a common spatial correlate of CD274 and IDO1 expression.

**Figure 7:**
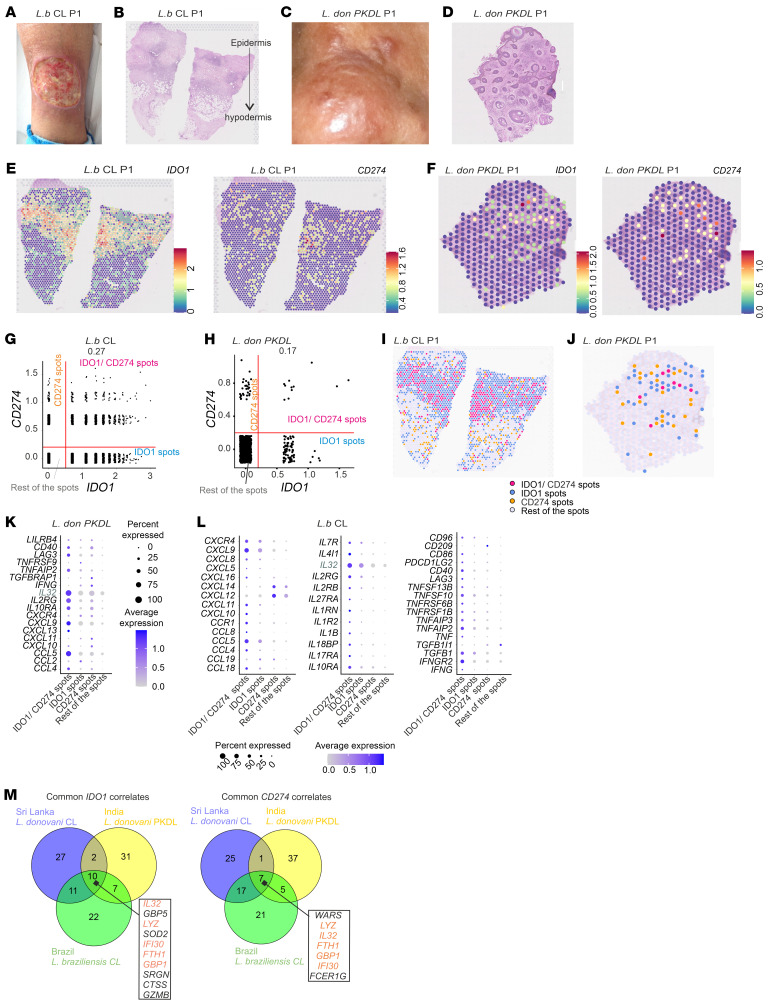
Visium spatial transcriptomics of IDO1 and CD274 niches in different dermal variants of leishmaniasis. (**A**) CL lesion caused by *L*. *braziliensis* in P1 from Brazil. (**B**) H&E image from **A**. (**C**) Polymorphic PKDL lesion caused by *L*. *donovani* in P1 from India. (**D**) H&E image from **C**. (**E** and **F**) IDO1 and CD274 spatial expression in *L. b*r*aziliensis* CL P1 (**E**) and *L. donovani* PKDL P1 (**F**). (**G** and **H**) *CD274* and *IDO1* expression scatter plots from all spots from *L*. *braziliensis*–infected CL skin lesions (*n* = 4) (see also Supplemental Table 2; thresholds: *x* = 0.5, *y* = 0.2) (**G**) and *L*. *donovani* PKDL P1 and P2 (**H**) (see also Supplemental Table 3; thresholds: *x*,*y* = 0.2). (**I** and **J**) Spatial plot of IDO1 and PD-L1 expression classes for *L. braziliensis* CL P1 (**I**) and *L. donovani* PKDL P1 (**J**). (**K** and **L**) Differential expression of cytokines, chemokines, ILs, and TNF- and IFN-related and checkpoint markers between IDO1 and CD274 classes in *L*. *braziliensis* CL (**K**) (*n* = 4) and PKDL (**L**) (*n* = 2). (**M**) Top 50 *IDO1*- and *CD274*-correlated genes overlap across SL CL (*n* = 6), *L*. *braziliensis* CL (*n* = 4), and *L*. *donovani* PKDL (*n* = 2). (See also Supplemental Tables 1–3). Original magnification, ×20 (**B**, **E**, and **I**) and ×21 (**D**, **F**, and **J**).

Although differing mechanisms of immunopathology may occur across the leishmaniasis disease spectrum, IDO1 and PD-L1 have been consistently identified in transcriptomics studies (20, 50–53). To assess whether the *CD274* and *IDO1* niche composition in Sri Lankan patients with CL was also present in other forms of dermal leishmaniasis, we examined biopsies from 4 Brazilian patients with *L*. (*V.*) *braziliensis* CL (Figure 7, A and B, Supplemental Figure 6, A and B, and Supplemental Table 2) and 2 patients from India with *Leishmania* *donovani* post kala-azar dermal leishmaniasis (PKDL) (Figure 7, C and D, Supplemental Figure 6, C and D, and Supplemental Table 3). Histologically, we observed a diffuse cellular infiltrate in these samples (Figure 7, B and D, and Supplemental Figure 6, B and D). Visium analysis indicated that *CD274* and *IDO1* mRNA similarly localized within the papillary dermis (Figure 7, E and F). Using the same analytical strategy (Figure 7, G and H), we found that *IDO1*^hi^*CD274*^hi^ spots were restricted mainly to the upper dermis (Figure 7, I and J). *IL32* mRNA was abundant in all IDO1/CD274 spots in both datasets, along with other cytokines (Figure 7, K and L, and Supplemental Figure 6, E and F). On the basis of the top 50 correlating mRNAs, we identified signatures associated with *IDO1* (*IL32*, guanylate-binding protein 5 [*GBP5*], granzyme B [*GZMB*], lysozyme [*LYZ*], superoxide dismutase 2 [*SOD2*], lysosomal thiol reductase [*IFI30*], ferritin heavy chain 1 [*FTH1*], guanylate-binding protein 1 [*GBP1*], serglycin [*SRGN*], cathepsin S [*CTSS*]) and CD274 (*IL32*, *LYZ*, tryptophanyl-tRNA synthetase [*WARS*], *IF130*, *FTH1*, *GBP1*, Fc-epsilon RI-γ [*FCER1G*]) across all 3 disease forms. A subset of 5 genes emerged as a core correlate across all 3 datasets, namely *IL32*, *LYZ*, *GBP1*, *IFI30*, and *FTH1* (Figure 7M).

### IL-32 is a predictive biomarker for the rate of cure in SL CL.

**Figure 8:**
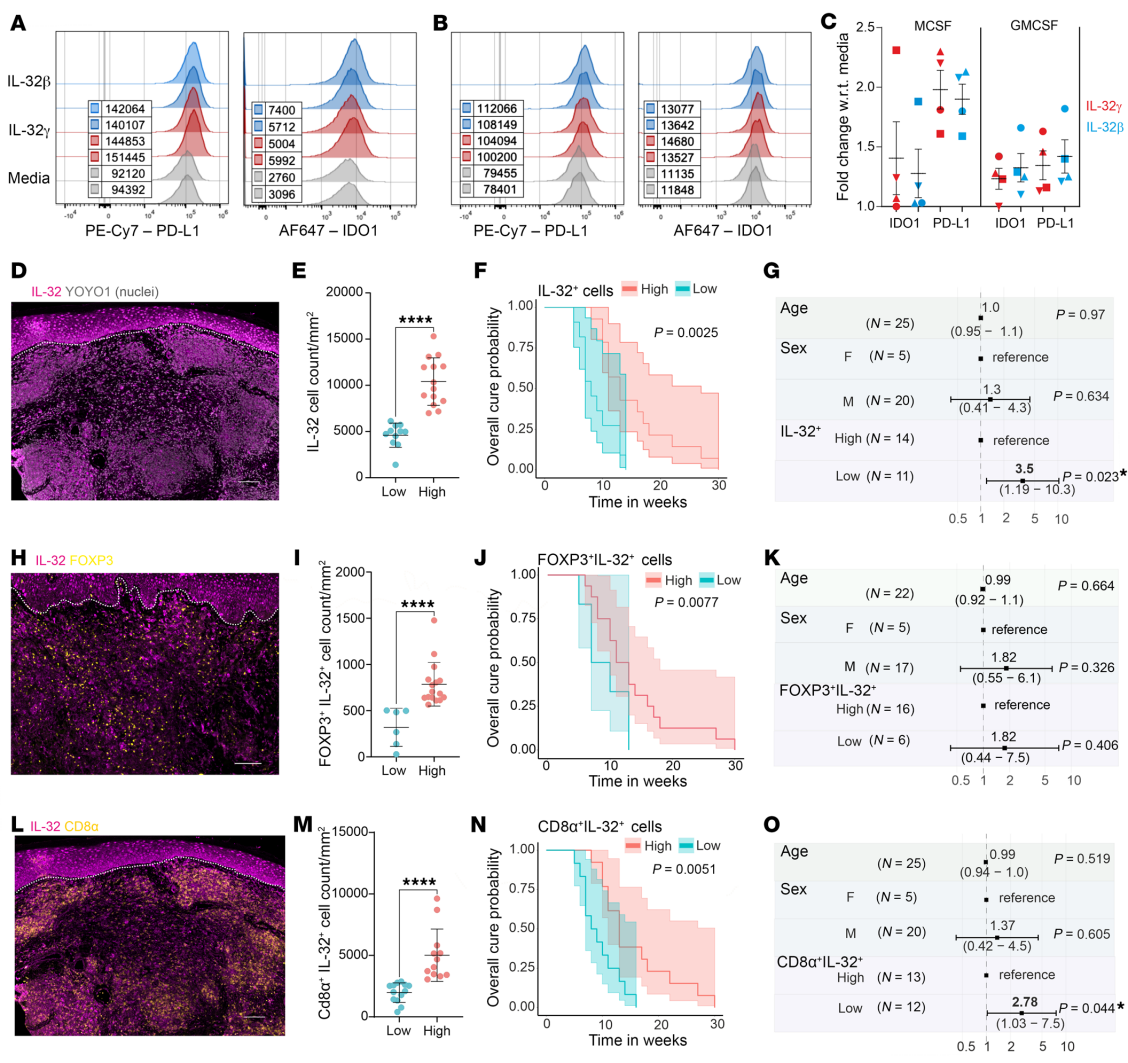
Protein analysis of IL-32-induced IC expression and prognostic value of lesional IL-32^+^ T cells for CL cure rate in Sri Lanka. (**A**) Histograms of IDO1 and PDL1 median fluorescence intensities in M-CSF–differentiated macrophages after IL-32γ (100 ng/mL) or IL-32β (50 ng/mL) stimulation. (**B**) Same analysis in GM-CSF–differentiated macrophages. (**C**) PDL1 and IDO1 expression fold changes in M-CSF and GM-CSF macrophages (*n* = 4). Symbols represent individual volunteers; colors indicate treatment. Data indicate the mean ± SEM. (**D**) IL-32 protein expression in SL CL. Scale bar: 100 μm. (**E**) Patient stratification by dermal IL-32^+^ cell density (*n* = 25; low = 11, high = 14). Data indicate the median. (**F**) Treatment response Kaplan-Meier plot for the IL-32 groups. Shaded areas = 95% CI. *P* value was determined by log-rank (Mantel-Cox) test. (**G**) Forest plot of a Cox hazard model (IL-32^+^ low vs. high), adjusted for age and sex, showing *n* values, HRs (95% CI), and *P* values. (**H**–**K**) Equivalent analyses for IL-32^+^FOXP3^+^ cells (*n* = 22). Scale bar: 100 μm. (**L**–**O**) Equivalent analyses for IL-32^+^CD8α^+^ cells (*n* = 25). Scale bar: 100 μm. Note: FOXP3^+^ and CD8α^+^ cell populations may include minor non-Treg and CD8^+^ T cell subsets. ****P < 0.0001, by 2-tailed Mann-Whitney *U* test (**E**, **I**, and **M**).

We differentiated peripheral blood monocytes with macrophage colony-stimulating factor (M-CSF) or GM-CSF to mimic in situ polarization, and then stimulated the cells with IL-32(β/γ) and measured IDO1 and PD-L1 expression by flow cytometry. IL-32(γ/β) stimulation increased surface PD-L1 expression in both GM-CSF– and M-CSF–polarized macrophages and increased intracellular IDO1 in GM-CSF–polarized macrophages. (Figure 8, A–C). This observation corroborates the reported capacity of IL-32 to upregulate these IC molecules (46, 47). Given the association between PD-L1 expression and cure rate upon treatment, we evaluated the relationship between IL-32 expression and treatment outcome. In contrast to our previous study that focused on altered IC molecule expression during therapy, here we examined pretreatment biopsies to determine whether IL-32 could inform treatment decisions. Quantitative analysis of IL-32 expression in the dermis of 25 SL CL patients (Figure 8D and Supplemental Table 1) allowed stratification into 2 groups (IL-32^hi^ or IL-32^lo^) based on the geomean number of IL-32^+^ cells/mm^2^ (Figure 8E). Patients who had IL-32^lo^ levels were significantly more likely to experience an early cure after treatment compared with patients with IL-32^hi^ levels (log rank test, *P* = 0.0025; Figure 8F), with an estimated age- and sex-adjusted Cox model HR of 3.5 (95% CI, 1.19–10.3, *P* = 0.023; Figure 8G). To determine whether this was due to IL-32^+^ Tregs, we stained tissue sections for IL-32 in combination with FOXP3 (*n* = 22 patients; Figure 8H). We found that 87% ± 4% (mean ± SD; *n* = 15 SL CL patients; Supplemental Table 1) of all FOXP3^+^ cells coexpressed CD3ε and CD4, supporting the use of FOXP3 to identify presumptive Tregs. Patients stratified on the basis of FOXP3^+^IL-32^+^ cell density (Figure 8I; *n* = 22 patients) did not show a significant difference in cure rate (Figure 8, J and K). In contrast, we found that patients with a low density of CD8^+^IL-32^+^ cells (*n* = 25 patients; Figure 8, L and M) were significantly more likely than those with a higher cell density to experience an earlier cure (log rank test, *P* = 0051; HR 2.78 (95% CI, 1.093–7.5, *P* = 0.044; Figure 8, N and O). Collectively, these data strongly argue that IL-32^+^CD8^+^ T cells are associated with the generation and/or maintenance of IDO1 and PD-L1 niches during CL and in such a way indirectly serve to restrain immune-dependent chemotherapy.

## Discussion

Enrichment of cells expressing IDO1 and PD-L1 is a common feature of chronic inflammatory diseases (54–58) and cancer (28, 59), underscoring the role of myeloid–T cell interactions in pathological TMEs. High-throughput spatial profiling studies have revealed immunosuppressive niches in cancer TMEs, comprising IDO1- and PD-L1–expressing suppressive macrophages, CD8^+^ T cells, and Tregs (21, 54, 60). Although 2 studies shed light on the regulators of IDO1 and PD-L1 in the TME (54, 61), they fell short of identifying the cellular and molecular drivers of this response.

Although the precise functions of IDO1 and PD1/PD-L1 in human leishmaniasis remain unclear, they are strongly associated with disease and treatment outcome. *Leishmania* increases expression of PD-L1 and IDO1 on antigen-presenting cells (20), and PD-L1/ PD-1 or IDO1 blockade leads to enhanced T cell proliferation, TNF-α and IFN-γ production, and reduced parasite load (16, 62–64). Patients with *L*. *braziliensis* infection have increased lesional expression of multiple IC molecules, and PD-1 blockade enhances antigen-specific T cell proliferation and IFN-γ production in vitro (65). Increased PD-1 and PD-L1 expression on T cells and monocytes is also associated with T cell dysfunction and disease progression in humans, dogs, and hamsters with visceral leishmaniasis (66–70) and PKDL (71). Hence, leishmaniasis provides a valuable infectious disease context in which to identify upstream cell and molecular regulators of IC molecule expression and their association with treatment outcome.

Our data on IDO1 and PD-L1 niches provide insights into the immunopathology of human CL. First, we identified myeloid cell–rich niches comprising mature DCs (DC3) and *CCL18*^+^ M2–like macrophages (CCL18 macrophages) in the papillary dermis with abundant *CD274* and *IDO1* mRNA levels. Second, neighborhood analysis identified close interactions between *CD274*^+^*IDO1*^+^ myeloid cells and CD8^+^ memory T cells and Tregs as well as *IDO1^–^CD274*^–^*CCL18*^+^ macrophages. Third, we identified a molecular signature associated with these niches (*CCL18*, *IL24*, *IL1B*, *TNFRSF6B*, *IFNGR2*, *CXCL9*, and *IL32*) and their cellular source. Finally, using geographically and clinically diverse forms of dermal leishmaniasis, we identified 5 common correlates of *IDO1* and *CD274* (*IL32*, *LYZ*, *GBP1*, *IFI30*, and *FTH1*). While both GBP1 (72) and FTH1 (73) have garnered interest as therapeutic targets in cancer and inflammation, IL-32 emerged as a key cytokine in our study. High abundance of IL-32^+^CD8^+^ T cells correlated with slower healing after SSG treatment, identifying these cells as a potential prognostic biomarker for treatment response.

Myeloid cells play a central role in the immunobiology of leishmaniasis and have been explored extensively in mouse models (74–76), but less so in human disease. Our immune landscape analysis formally demonstrates myeloid cell complexity in CL lesions, in terms of phenotypic heterogeneity and spatial organization. We highlight 2 notable findings. First, monocyte-derived DCs (moDCs) and DCs with abundant *IDO1* and *CD274* mRNA expression were extensively distributed in the lesion core and T cell–rich regions in the hypodermis, supporting the notion from data on other chronic inflammatory conditions that DCs with a regulatory phenotype might contribute to local immunosuppression (26, 77). Second, the lesion core contains abundant CCL18-expressing macrophages, also with abundant *IDO1* and *CD274* mRNA expression. CCL18 helps recruit naive T cells (78) and Tregs (36) and, in a fashion analogous to CCL18^+^ tumor-associated macrophages (TAMs) in cancer (79), may promote a regulatory environment. *IDO1*^+^*CD274*^+^ *CCL18*^+^ macrophages also express *IL1B*, *IL24*, and *IFNGR2*, suggestive of macrophage activation by both IFN-γ and TNF (27, 30, 80). This conclusion is supported by coexpression of *TNF* and *IFNG* in IDO1^+^CD274^+^ spots in the lesion core, associated with T cell infiltration. Thus, in CL, the regulation of IC molecules appears distinct from that recently described in the IFN-γ–depleted core of tuberculosis granulomas (23).

A central finding of this study was the identification of IL-32 as a core component of the IDO1/PD-L1 niche in multiple forms of dermal leishmaniasis. IL-32 is a complex and still poorly understood cytokine that can be produced by multiple cell lineages (81). Studies of IL-32 are hampered by multiple isoforms, each with distinct biological effects, and the lack of IL-32 in rodents (82). Both IL-32ß and IL-32γ are proinflammatory, with the β isoform being the most abundant and the γ isoform being the most biologically active (83). IL-32 has been mooted as a biomarker in skin conditions including atopic dermatitis and melanoma (84) and ascribed a proinflammatory, host-protective role in tuberculosis, leishmaniasis, colitis, and arthritis on the basis of the use of human *IL32*–transgenic (hu*IL32*-transgenic) mouse models. Enhanced lesional expression of IL-32 was also noted in patients with *L*. *braziliensis* infection (49, 82). We confirmed high expression of both IL-32γ and IL-32ß in our patient biopsies, identified the cellular sources of lesional IL-32, confirmed that IL32β/γ upregulated IC molecule expression in myeloid cells, and demonstrated that IL-32^+^CD8^+^ T cells represent a key molecular and cellular component of the IDO/PD-L1 niche. In keeping with other facets of IL-32 biology, our data imply a microenvironment- and context-dependent immunoregulatory role for IL-32.

Despite having different pathologies, our analysis revealed a core gene signature (*IL32*, *LYZ*, *GBP1*, *IFI30*, and *FTH1*) associated with *IDO1* and *CD274* expression across multiple forms of dermal leishmaniasis. In contrast to IL-32, which was mainly associated with CD8^+^ T cells and Tregs, *LYZ*, *GBP1*, *IFI30*, and *FTH1* are all prototypical myeloid cell markers. Further studies are required to determine whether these other members of this core signature contribute to shaping the immunoregulatory environment.

In our previous study of CL, we identified elevated expression of IDO1 and PD-L1 in pre-treatment compared with on-treatment biopsies and demonstrated that an early reduction in PD-L1 after treatment onset was predictive of the rate of cure (20). Although providing a mechanistic basis for how ICs might restrict the efficacy of T cell–dependent chemotherapy, this earlier study did not provide insights into the cellular or molecular mechanisms responsible for driving IC molecule expression. Our current data derived from spatial interaction mapping and validated through multiple orthogonal approaches allow us to now propose a model for the regulation of IC molecules: (a) CCL18^+^ macrophages recruit IL-32^+^CD8^+^ T cells and IL-32^+^ Tregs into a myeloid cell niche; (b) IL-32^+^CD8^+^ T cells and IL-32^+^ Tregs induce the expression of IC molecules in DCs and macrophages by paracrine signaling through the as-yet-uncharacterized IL-32 receptor; (c) IDO1^+^PD-L1^+^ myeloid cells respond to T cell–derived cytokines (IFN-γ, TNF) and secrete additional M2-polarizing cytokines such as IL-24 and IL-1β; and (d) IDO1 and PD-L1 expressed by DCs and macrophages in the niche promote loss of effector T cell function through tryptophan metabolism, production of biologically active kynurenine and other downstream metabolites, and PD-1 signaling, respectively.

The study has some limitations. On ethical grounds, only 2 biopsies were permissible, precluding the measurement of IDO1 and PD-L1 levels upon complete disease resolution. Patients with low cell density of IL-32^+^CD4^+^FOXP3^+^ presumptive Tregs showed a trend toward faster healing that may have been more evident with a larger sample size. Challenges associated with the field collection and processing of samples in a remote cutaneous leishmaniasis–endemic setting limited our ability to perform phenotypic and/or functional characterization of cells beyond that possible with FFPE blocks. We cannot, therefore, formally address questions related to T cell antigen specificity or apply deeper phenotyping methods (e.g., Cite-Seq) to extend our analysis to nonconventional CD8αβ^+^/CD8α^+^ cells, including mucosal-associated invariant T (MAIT) cells and TCRγδ cells. Finally, we limited our current analysis to exploring cell-extrinsic regulation of IDO1 and PD-L1 on myeloid cells. Ongoing studies are exploring cell-intrinsic regulation directly to intracellular parasitism by *Leishmania* (unpublished observations).

In conclusion, given the commonality of niche composition across multiple forms of dermal leishmaniasis, it is tempting to speculate that our observations may also contribute to local immunoregulation and treatment response in multiple forms of dermal leishmaniasis and that similar regulation of the IDO1/PD-L1 niche may occur during other infectious and noninfectious diseases.

## Methods

### Sex as a biological variable.

We analyzed biopsies from both sexes and included sex as a variable in Cox proportional hazard models examining T cell associations with cure rates (see Supplemental Tables 1–3).

### Patient cohorts.

We studied 3 treatment-naive cohorts: SL CL (*n* = 25, *L*. *donovani*) (20); Brazilian CL (*n* = 4, *L*. *braziliensis*); and Indian PKDL (*n* = 2, *L*. *donovani*). For clinical metadata, see Supplemental Tables 1–3.

### Visium spatial transcriptomics.

FFPE blocks were first cooled, and then 5 μm serial sections cut onto Superfrost slides (Thermo Fisher Scientific) and processed using Visium Spatial gene expression kits (10x Genomics). Slides were deparaffinized, H&E stained, and then scanned (Axioscan). After de-crosslinking and overnight human probe hybridization, libraries were prepared per the manufacturer’s protocol and sequenced (NovaSeq 6000). FASTQ files were aligned to GRCh38 using Spaceranger_1.3.0 to generate UMI counts per spatial barcode, followed by normalization. For Visium data analysis, see Supplemental Methods. For analysis code, see *Data availability*.

### CosMx single-cell spatial transcriptomics.

FFPE samples from 4 SL CL patients (P3–P6; Supplemental Table 1) were analyzed at NanoString Translational Services using the CosMx Human Universal Cell Characterization RNA Panel (1,000-plex) under the Technology Access Program (TAP). For the CosMx data analysis pipeline, see Supplemental Methods.

### Human monocyte–derived macrophage culturing and stimulation.

CD14^+^ monocytes were purchased form Charles River Laboratories (PB14C-2; *n* = 1) or isolated from National Health Service (NHS) blood cones (DB202111; *n* = 3) and differentiated with M-CSF (Proteintech, HZ-1192) or GM-CSF (Proteintech, HZ-1002) in complete media for 6 days, refreshed on day 3. On day 7, human monocyte–derived macrophages were stimulated with IL-32β (50 ng/mL, R&D Bio-Techne, 6769-IL-025) or IL-32γ (100 ng/mL, R&D Bio-Techne, 4690-IL-025/CF) for 24 hours. Cells were analyzed for IDO1 and PD-L1 expression by flow cytometry using FlowJo, version 10.10. The antibodies used are listed in Supplemental Methods.

### Immunostaining and image analysis.

FFPE sections from 23 of 25 Sri Lankan patients (see *Patient cohorts*) were analyzed. The protocols and antibodies used are listed in Supplemental Methods. Tissue sections were scanned using a Zeiss Axioscan.Z1 (×20 magnification) and analyzed with StrataQuest, version 7.0.1.178. For nuclear segmentation, YOYO1 staining was performed, with cell polygons created for CD8 (outside), PD-L1, and IDO1 (outside/inside). IDO1^+^PD-L1^+^ cells were identified using intensity thresholds based on visual inspection. Proximity maps at 25, 50, and 100 μm from IDO1^+^, PD-L1^+^, or IDO1^+^PD-L1^+^ double-positive cells were generated to detect CD8^+^ cells. For IL-32 and CD8/FOXP3 analysis, nuclear masks were expanded by 10 μm for IL-32 and by 1 μm for CD8 (ring mask: –0.20/^+^0.20 μm). For FOXP3 analysis, nuclear masks without expansion were used. Standardized intensity thresholds were applied across all samples. IL-32^+^CD8^+^ and IL-32^+^FOXP3^+^ cells were identified using scattergraph quadrant analysis.

### IL-32 PCR.

See Supplemental Methods for details.

### Statistics.

For statistical analyses, we used GraphPad Prism (GraphPad Software) with 2-tailed, nonparametric tests (Mann-Whitney *U* or Kruskal-Wallis), applying Dunn’s adjustment for multiple comparisons where needed. Significance was set at a *P* value of less than 0.05. Data represent the mean ± SD unless otherwise indicated in the figure legends. Details of sample selection and multivariate Cox proportional hazards model are provided in Supplemental Methods.

### Study approval.

Written informed consent was obtained from all participants for inclusion and use of photographs. The study was approved by the ethics committees of the University of Jayewardenepura (780/13 and 52/17), the Universidade de Sao Paulo (CAAE 39964520.8.0000.0068), the School of Tropical Medicine and IPGME&R Kolkata (IPGME&R/IEC/2019/208), and the University of York (PK201805) and were conducted following Declaration of Helsinki (2013) guidelines. Use of NHS blood cones was approved by the Biology Ethics Committee of the University of York.

### Data availability.

Raw sequencing data are deposited in the NCBI Gene Expression Omnibus (GEO) database (GEO GSE290027). Processed data are available on Zenodo (https://doi.org/10.5281/zenodo.10402126). Analysis code and figure generation scripts are available at GitHub (https://github.com/NidhiSDey/leish-ME/commit/a791158; commit ID: a791158). All graphical data values are provided in the Supporting Data Values file.

## Author contributions

NSD, SD, and PMK conceptualized the study. NSD developed the methodology. Experiments were conducted by NSD, SD, NB, SJ, LG, JALL, LCR, RS, and DB, while NSD and SD performed data analysis. PMK, SR, HG, and MC acquired funding, with PMK supervising the research. The original draft was written by NSD and SD, with review and editing by all authors.

## Supplementary Material

Supplemental data

Supporting data values

## Version 1 05/15/2025

Electronic publication
