# Supplementary material for: IL-32–producing CD8+ memory T cells define immunoregulatory niches in human cutaneous leishmaniasis
Source: J Clin Invest. 2025 May 15;135(10):e182040. doi: 10.1172/JCI182040 (PMC12077899; doi:10.1172/JCI182040)
Supplement: Supplemental data [file jci-135-182040-s180.pdf]

Interleukin-32 producing CD8+ memory T cells define immunoregulatory niche in human cutaneous leishmaniasis

Authors:

Nidhi S. Dey[1], Shoumit Dey[1], Naj Brown[1], Sujai Senarathne[2], Luiza Campos Reis[3, †], Ritika Sengupta[4], Jose Angelo L. Lindoso[5,6], Sally James[7], Lesley Gilbert[7], Mitali Chatterjee[4], Hiro Goto[3], Shalindra Ranasinghe[2] and Paul M. Kaye[1*]

Affiliations:

[1]York Biomedical Research Institute, Hull York Medical School, University of York, UK.

[2]Department of Parasitology, Faculty of Medical Sciences, University of Sri Jayewardenepura, Sri Lanka.

[3]Department of Preventive Medicine, Instituto de Medicina Tropical de São Paulo, Faculdade de Medicina, Universidade de São Paulo, Brazil.

[4]Department of Pharmacology, Institute of Postgraduate Medical Education and Research, Kolkata, India.

[5]Secretaria de Saúde do Estado de São Paulo, Instituto de Infectologia Emílio Ribas, São Paulo, SP, Brasil

[6]University of São Paulo, Faculty of Medicine, Department of Infectious and Parasitic Diseases, São Paulo, SP, Brazil.

[7]Technology Facility, Department of Biology, University of York, UK

*Corresponding author. Email: paul.kaye@york.ac.uk

[†] Present address: Escuela Profesional de Medicina Humana, Facultad de Medicina, Universidad Nacional Toribio Rodríguez de Mendoza de Amazonas, Chachapoyas, Peru.

This Supplementary material contains:

1. Supplementary Methods

2. Supplemental Figure 1 to 6.

3. Table S1 to S3 supplementary tables

Supplementary methods

Study design.

This study analyzed lesion microenvironments, cellular neighborhoods, and cytokine regulation of immunosuppressive IDO1+/PD-L1+ myeloid cells across different geographical manifestations of human cutaneous leishmaniasis. Spatial transcriptomics, though limited in sample size, provided high-throughput profiling of hundreds to thousands of spots and cells per tissue section. Data collection in endemic countries and analysis at UoY were conducted independently, with clinical data unavailable until analysis completion, ensuring blinded evaluation of immune status. Biological replicates were included in spatial transcriptomics. For IHC, 2-3 serial sections per patient were analyzed, with experiments repeated 2-3 times. qPCR experiments included duplicate reactions and were repeated thrice.

Sample selection for analyses

Sample selection was based on tissue quality, availability, parasite presence, and clinical outcome. From Sri Lankan cohort (n=25), 23 samples were analyzed (2 excluded: insufficient tissue), with 22 used for IL-32/FOXP3 analysis (1 tissue detachment). Six Amastin+ samples underwent 10x Visium analysis, four selected for CosMx based on parasite load. From Brazilian cohort, four of 12 PCR/ELISA-positive samples (from 20 screened) were selected for 10x Visium, representing two unhealed (3-month score >0) and two healed cases. Two Indian PKDL samples were chosen for 10x Visium based on histopathology and RNA quality. Details in Supplemental Tables 2-3.

Multivariate Cox proportional hazard model

Patient samples (n=22-25) were analyzed for IL-32^{+}, CD8^{+} IL-32^{+}, and FOXP3^{+} IL-32^{+} cells/mm^{2} and stratified by geomean thresholds into high/low groups: IL-32 (>6969.37, n=14;

<6969.37, n=11), $CD8^{+}IL\text{-}32^{+}$ (>2800.9, n=13; <2800.9, n=12), and $FOXP3^{+}IL\text{-}32^{+}$ (>544.0, n=16; <544.0, n=6). Kaplan-Meier survival curves were generated using survfit and survdiff functions (survminer, survival, ggplot2 packages). Multivariate hazard models, adjusted for age and sex, were constructed using survival and survminer packages, with hazard ratios derived from Wald's statistic and visualized on forest plots.

Patient diagnostic methods

Slit skin smears (SSS) and punch biopsy: For Sri Lankan cohort method used is described previously(1) and similar methods were followed for Indian and Brazillian cohorts.

PCR: DNA extraction was performed from 2mm excision biopsy and was used as an input material for diagnostic PCR for *Leishmania* positivity as previously described (1) .In Sri Lanka, LITSR/L5.8S specific primers were used that amplifies a 320 bp fragment of ITS1 region of Leishmania genus-specific DNA. In Brazil, kDNA specific primers were used that amplify 750 bp kinetoplastid DNA of *Leishmania* subgenus *Viannia* (2). In India, DNA extraction was performed using manufacturer's instructions using QIAamp DNA mini kit, Qiagen, Hilden, Germany from a skin biopsy collected in phosphate- buffered saline (20 mM, pH 7.4) and eluted in 50 µL of DNA elution buffer. Real-time PCR was performed using specific primers for kinetoplast minicircle gene, using 1ul of DNA as input material and 400 nM of each primer. DNA isolated from a *Leishmania donovani* strain MHOM/IN/1983/AG83 served as the positive control while negative controls used were DNA from a healthy donor (no amplification), and a reaction mixture with water instead of template DNA (no-template control) [(3)].

Diagnostic PCR primers for *L. don* CL	LITSR (forward) 5'-CTGGATCATTTTCCGATG-3	L 5.8S (reverse) 5'-TGATACCACTTATCGCACTT-3'
Diagnostic PCR primers for *L. (V)* kDNA	B1. 5'−GGGGTTGGTGTAATATAGTGG−3'	B2. 5'−CTAATTGTGCACGGGGAGG−3'
Diagnostic PCR primers for *L. don.* PKDL	Forward: 5'-CTTTTCTGGTCCTCCGGGTAGG-3'	reverse: 5'-CCACCCGGCCCTATTTTACACCAA-3'

ELISA and Indirect immunofluorescence reaction: In Brazil, all sera were tested for immunoglobulin G (IgG) antibodies at 1:50 dilution in the ELISA using whole *L. major*-like parasite lysate (ELISA–*L. major*-like) as previously described (4). For both antigens, the cut-off point was determined using a receiver operating characteristic (ROC) curve. The reactivity index (RI) was calculated for each sample by dividing the sample absorbance value by the cut-off. Samples were considered positive if the RI value was ≥ 1. The spectrophotometric reading of each well was performed at 450 nm using a Multiskan GO instrument (Thermo Scientific, Finland). The IIF test was performed on the slides, containing the suspension of fixed promastigotes of *Leishmania* (*Leishmania major*-like promastigotes/MHOM/BR/71/49) as

previously described (5). Then the slide was observed in the fluorescence microscope, with the objective of 250 ×.

Immunostaining

The staining protocol involved following steps: heat fixation at 60°C to ensure sample adherence, followed by two rounds of 5-minute deparaffinization in histo clear/xylene at room temperature (RT). Subsequent steps included equilibration in 95% ethanol for 3 minutes, 70% ethanol for another 3 minutes, and hydration in distilled water for 3 minutes. Antigen retrieval was achieved through a 15-minute high-pressure treatment with citrate buffer (pH 6.0), followed by a 25-minute standing period at RT. Two 5-minute washes with wash buffer (PBS + 0.5% BSA w/v were followed with blocking (PBS + 0.1% Triton X-100 v/v + 5% Normal donkey serum v/v + 5% Normal goat serum v/v + 1% BSA w/v) for 30 minutes, overnight primary Ab incubation with IDO1 (1:200) and PD-L1 (1:500) at 4°C in the fridge, and three 5-minute washes the next day. Secondary ab staining for 30-45 minutes at RT employed CF750 Donkey Anti-Rabbit IgG CF™ 750 and Goat anti-Mouse Dy650 at a 1:500 concentration. Sections were then incubated with CD8-AF594 for 2h at room temperature. After three more buffer washes, YOYO-1 at 0.2uM concentration was added for 30 minutes in wash buffer followed by three more wash buffer washes and subsequent mounting in Prolong Gold mounting media. For CD3e CD8b set, sections were incubated with CD3 (1:300) and CD8b (1:1000) overnight at 4°C in the fridge and were developed in CF750 Donkey Anti-Rat IgG CF™ 750 and Goat Anti-Mouse IgG H&L (Alexa Fluor® 647; preadsorbed) for 1 h at RT the next day followed by CD8a-AF594 (1:100) incubation for 1h at RT. For CD3, CD4 and FOXP3 set, both CD3 and FOXP3 (1:400) were incubated overnight, developed in Donkey Anti-Rat CF750 and Goat anti mouse AF555 F(ab)2, followed by CD4-AF488 (1:100) for 1 h at RT, the next day.

For the IL-32 CD8 set and IL-32 FOXP3, antigen retrieval utilized TE buffer (10mM Tris Base, 1mM EDTA, 0.05% Tween 20, pH 9.0), while pre-primary blocking consisted of PBS + 0.1% Triton X-100 v/v + 5% Normal goat serum v/v + 1% BSA w/v. The slides were incubated with IL-32 (1:400) and CD8a or FOXP3 overnight at 4°C and were developed with goat anti-rabbit Dylight 650 and Goat anti-mouse AF555 F(ab)2 at a 1:500 concentration, respectively.

To ascertain specificity of CD8α+ and FOXP3 cells, tissue sections were stained with either CD3ε and CD8α (n=23) in one set or CD3 ε, CD4 and FOXP3 (n=15 SL CL patients) in another set.

**Antibody**	**Catalogue No.**	**clone**	**Supplier**	**Dilution**
Rabbit anti human IL32	11079-1-AP	Polyclonal	Proteintech	1:400
Rabbit anti human IDO1	86630	D5J4E	CST	1:200
Alexa Fluor® 594 anti-human CD8a Antibody	372904	C8/144B	Biolegend	1:100
Purified anti-human CD8a [C8/144B] 100 ug	372902	C8/144B	BioLegend	1:100
CD8b (4B3B8) human purified	68432-1-IG-20UL	4B3B8	Proteintech	1:1000

Mouse anti-human PD-L1 mAb	29122	405.9A11	CST	1:400
Recombinant mouse Anti-FOXP3 antibody	ab20034	236A/E7	abcam	1:500
Anti-CD3 antibody [CD3-12] (ab11089)	ab11089	CD3-12	Abcam	1:300
CD4-AF488	ab196372	EPR6855	abcam	1:100
IgG goat anti rabbit Dylight 650	SA5-10034		Invitrogen	1:500
Goat anti mouse AF555 F(ab)2	A21425		Invitrogen	1:500
Donkey Anti-Rabbit IgG CF™ 750	20298		Biotium	1:500
Donkey Anti-Rat CF750	20857-50uL		Biotium	1:500
YOYO-1	Y3601		Invitrogen	1:500
DAPI	D9542-1MG		Sigma Aldrich	1:5000

Flow antibodies used in the study

Antibody	Catalogue No.	clone	Supplier	Dilution
Anti-IDO1 Mouse Monoclonal Antibody (Alexa Fluor® 647)	654004	2E2/IDO1	Biolegend	1:20
CD274 PE-Cy7 (B7-H1, PD-L1) human	329718	29E.2A3	BioLegend	1:20
BV650 mouse anti-human CD14	563419	M5E2	BD Biosciences	1:20
Zombie Aqua™ Fixable Viability Kit	423102		BioLegend	1:1000

Visium data integration and clustering

Spot matrices were analyzed using Seurat_4.3.0 in R 4.2.3. After SCTransform normalization, samples were integrated using FindIntegrationAnchors() and IntegrateData() based on 3000 features. Clustering was performed using 30 principal components at 0.5 resolution, with UMAP visualization. Differential expression (DE) analysis used PrepSCTFindMarkers() for depth normalization, followed by FindAllMarkers() (log2fold

change >0.25, min.pct=0.25). DE significance used Wilcoxon Rank Sum tests. CellMesh(6) identified probable cell types per cluster. Results were visualized using SpatialDimPlot, EnhancedVolcano, and SpatialPlot() for IDO1 and CD274 expression. Comparison across My1, My2 and My3 clusters used violin plots in Graphpad..

Deconvolution of Visium spots to identify immune cell abundance

Cell2location was used to deconvolve cell types in 55µm Visium spots (N_cells_per_location=30, detection_alpha=20), using Reynolds et al. skin single-cell data (E-MTAB-8142; www.ebi.ac.uk/arrayexpress/experiments/E-MTAB-8142) as reference. Cell-type signatures were estimated using negative binomial regression (1,000 epochs), retaining Reynolds et al. cell-type annotations. This was followed by spatial data deconvolution (30,000 epochs) using the reference signature model. Cell abundance was inferred using 5% quantile values of posterior distribution, then analyzed across My1-3, IDO1, CD274, and IDO1/CD274 spots.

Identifying molecular networks associated with CD274 and IDO1 expression

IDO1 and CD274 correlation across tissue spots from Sri Lankan patients (n=6) was visualized using Seurat's FeatureScatter(). Spots were classified using median thresholds (IDO1=1.2, CD274=0.6) into four categories: IDO1 (IDO1>1.1, CD274<0.5), CD274 (IDO1<1.1, CD274>0.5), IDO1/CD274 (both >1.1, >0.5), or Rest of the spots (both <1.1, <0.5). DE genes were visualized using GraphPad 9.3.1 (line plots) and Seurat's DotPlot. This analysis was replicated for Brazilian CL (n=4) and Indian PKDL (n=2) samples.

Pairwise gene correlations with IDO1 and CD274 were calculated using Spearman's method (R stats package, p<0.05). Top 50 correlated genes from each cohort were compared using

Venny 2.0 (https://bioinfogp.cnb.csic.es/tools/venny/index2.0.2.html) to identify common correlates across datasets.

Single cell resolution spatial transcriptomic mapping of CL lesions using CosMx single molecular imager.

Twenty FOVs were analyzed across sections derived from four patients. Data was integrated using the Giotto package (9). Cells were filtered (minimum expression threshold=1, >5 features/cell, >5 cells/feature), normalized for library size (scaling factor=6000), log-transformed, and z-scored. Expression values were regressed for feature expression sum and features per cell to account for batch effects.

Clustering and cell type annotation of CosMx data

Cell data was visualized using UMAP, with quality control for count-based clustering bias. NanoString's nb_clust algorithm identified 16 immune cell types, while 6 additional unsupervised clusters (KC1-3, plasmablast2, fib2, mac2) were annotated using CellMesh (6) and Azimuth PBMC Single cell sequencing and CITE-seq reference data (10). Differential expression between clusters used findMarkers_one_vs_all (Gini coefficient) and plotMetaDataHeatmap. Further subtyping in Seurat identified 13 myeloid subtypes from mac, mac2, mDC, neutrophil, pDC, monocyte, and mast cells. Cells with border transcript contamination (12%) were excluded. Cell types were spatially mapped using ImageDimplot (Seurat) and SpatPlot (Giotto).

Neighborhood analysis of *CD274* and *IDO1* expressing cells in CosMx dataset

Myeloid cells were classified as IDO1mye+ (*IDO1* > 3, *CD274* < 3), CD274mye+ (*IDO1* < 3, *CD274* > 3), or IDO1+/CD274mye+ (*IDO1* > 3, *CD274* > 3). Giotto's SpatPlot2D mapped cell types spatially. Spatial networks (createSpatialNetwork) were created using Giotto (4 nearest neighbors based on plotStatDelaunayNetwork) to map interactions between these populations and their immediate neighbors. Cells were categorized as "source," "neighbor," "both," or "others." UpsetR shiny app (11) and manual validation estimated neighbor combinations. Expression of cytokines and chemokines in source cells and top 5 neighbors (CCL18_mac, Treg, IDO1mye+, CD274mye+, T CD8 memory) was analyzed using violin plots, heatmaps (Giotto), and bar charts (GraphPad Prism 10).

IL-32 PCR

RNA from FFPE samples was extracted using RNeasy FFPE Kit (Qiagen), eluted in 14-30 µl, and quantified by NanoDrop. cDNA was synthesized from 50ng RNA using Superscript IV with random hexamers (Invitrogen). qRT-PCR was performed in duplicate using Fast SYBR Green on StepOnePlus (ThermoFisher), with LPS-stimulated PMA-differentiated THP-1 cells as positive controls for IL-32 levels (12). Transcript levels were calculated by ΔΔCt method using GAPDH/ACTB reference genes and healthy skin controls. Primers are listed in supplementary materials.

IL32 primers used in the study

IL32alpha	GCTGGAGGACGACTTCAAAGA
IL32alpha	GGGCTCCGTAGGACTTGTCA
IL32beta	CAGTGGAGCTGGGTCATCTCA
IL32beta	GGGCCTTCAGCTTCTTCATGTCATCA
IL32gamma	AGGCCCGAATGGTAATGCT
IL32gamma	CCACAGTGTCCTCAGTGTCACA

Supplementary figures:

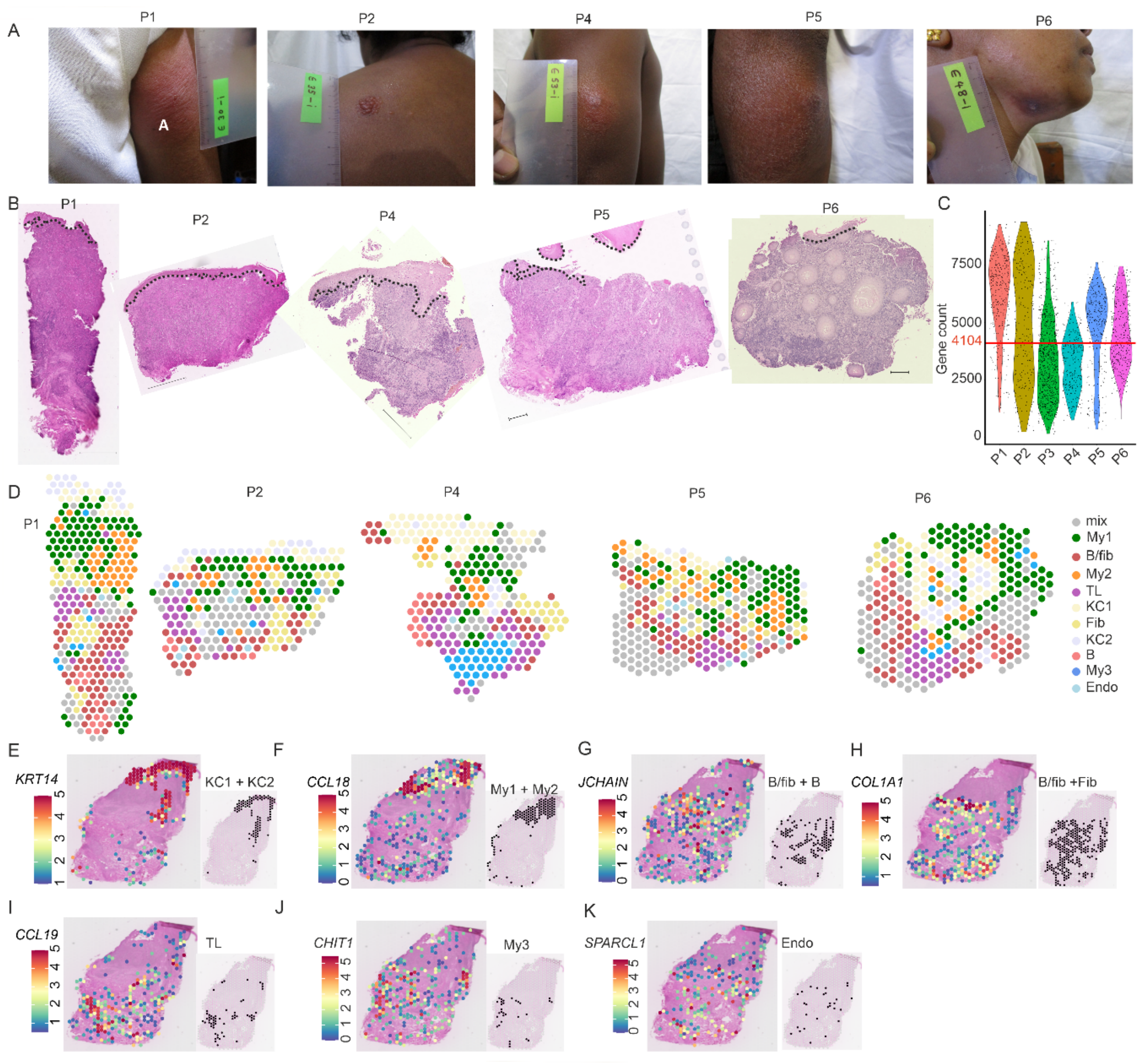

Supplemental Figure 1 10x Visium spatial transcriptomics on *Leishmania donovani* infected CL skin.

(A) Photographs of lesions in patients P1, P2, P4, P5 and P6. **(B)** H&E images of patients described in (**A**) showing dense cellular infiltrate. **(C)** Violin plot showing overall gene counts across all patients. Red line shows median gene count across all samples (n=6) **D**, Spatial maps for patients described in (**A)** coloured by clusters identified in Figure 1K. **E-K,** Spatial feature plot for P3 overlaid on H&E showing normalised gene expression of top genes expressed in each cluster: *KRT14*, *CCL18*, *JCHAIN* , *COL1A1* , *CCL19* , *CHIT1* and *SPARCL1* (left panels) and spatial maps of respective clusters (right panels).

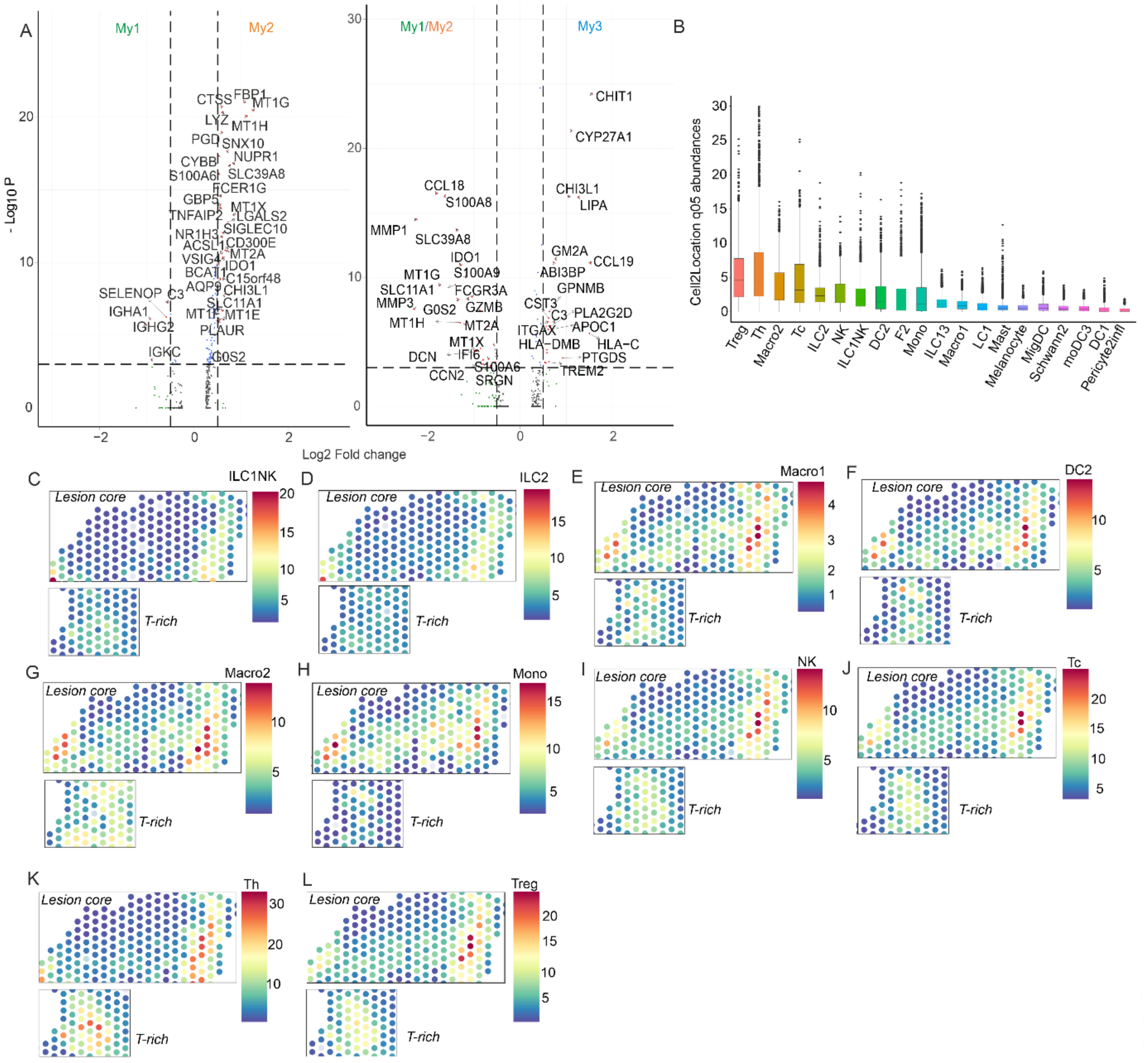

Supplemental Figure 2: DE between My1 and My1 and deconvolution of cell types in 10x Visium spots

(**A**) Volcano plot showing differentially regulated genes in My1 vs. My2 spots (left panel) and in My1/My2 vs. My3 spots. p-values are thesholded at -Log_{10}P value of 3 and Log2Fold change of -0.5 and 0.5. N=6 SL CL patients. (**B**) Bar plot showing combined 5th quantile predicted cell abundances per spot for P1-P6. (Black line, median; bar, IQR). **(C-L)** Spatial plots of predicted abundance of cell types in lesion core and T-rich regions (shown for patient P3).

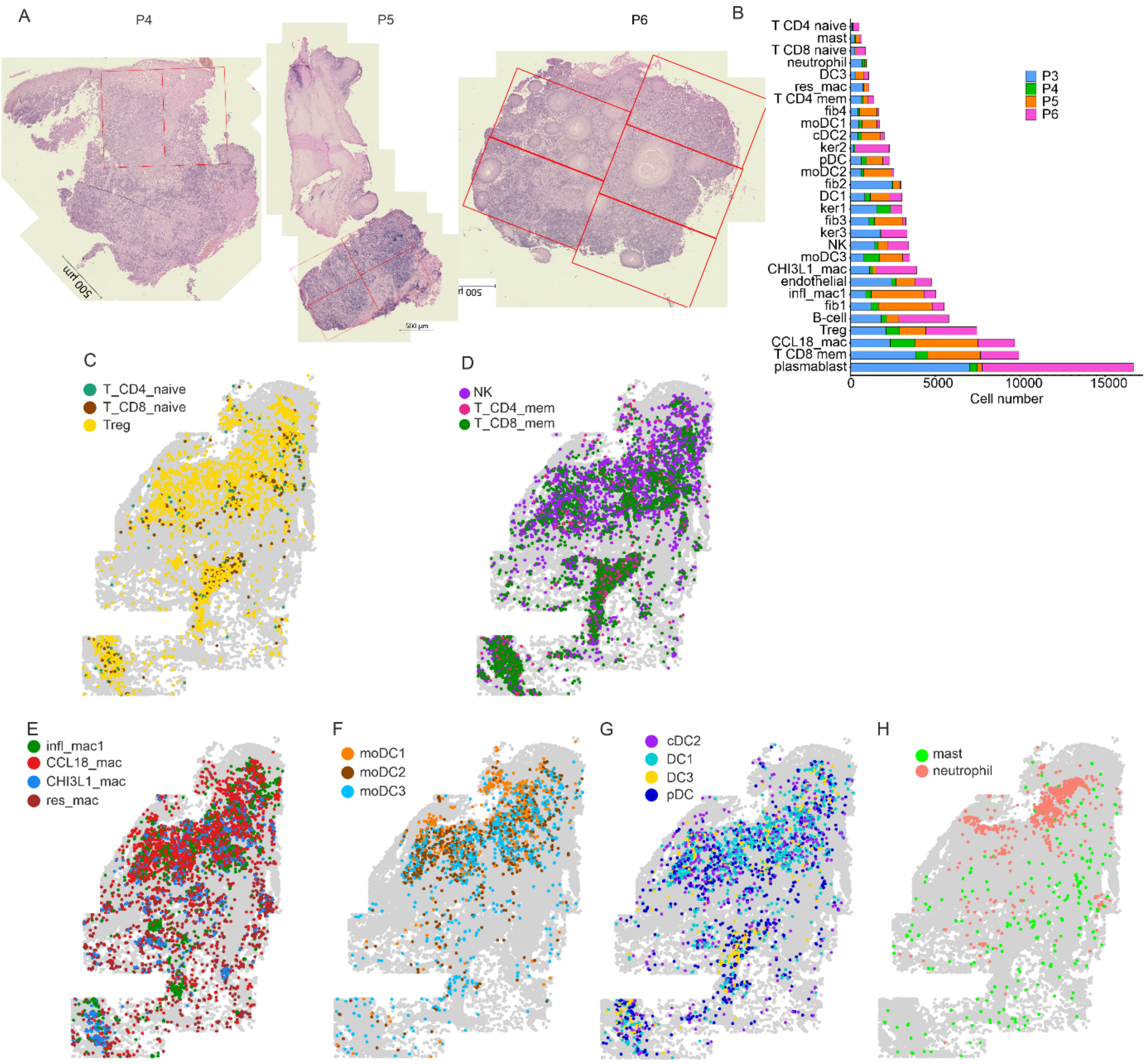

Supplemental Figure 3: CosMx FOV positions, spatial expression and cell number per cluster

(A) H&E images for patients P4-P6 showing FOV analysed using Nanostring CosMx. Bar 500 µm. (**B**) Stacked bar plot showing total cell counts across all patients for each population. (**C-H**) Single-cell spatial mapping of indicated cell types for patient P3

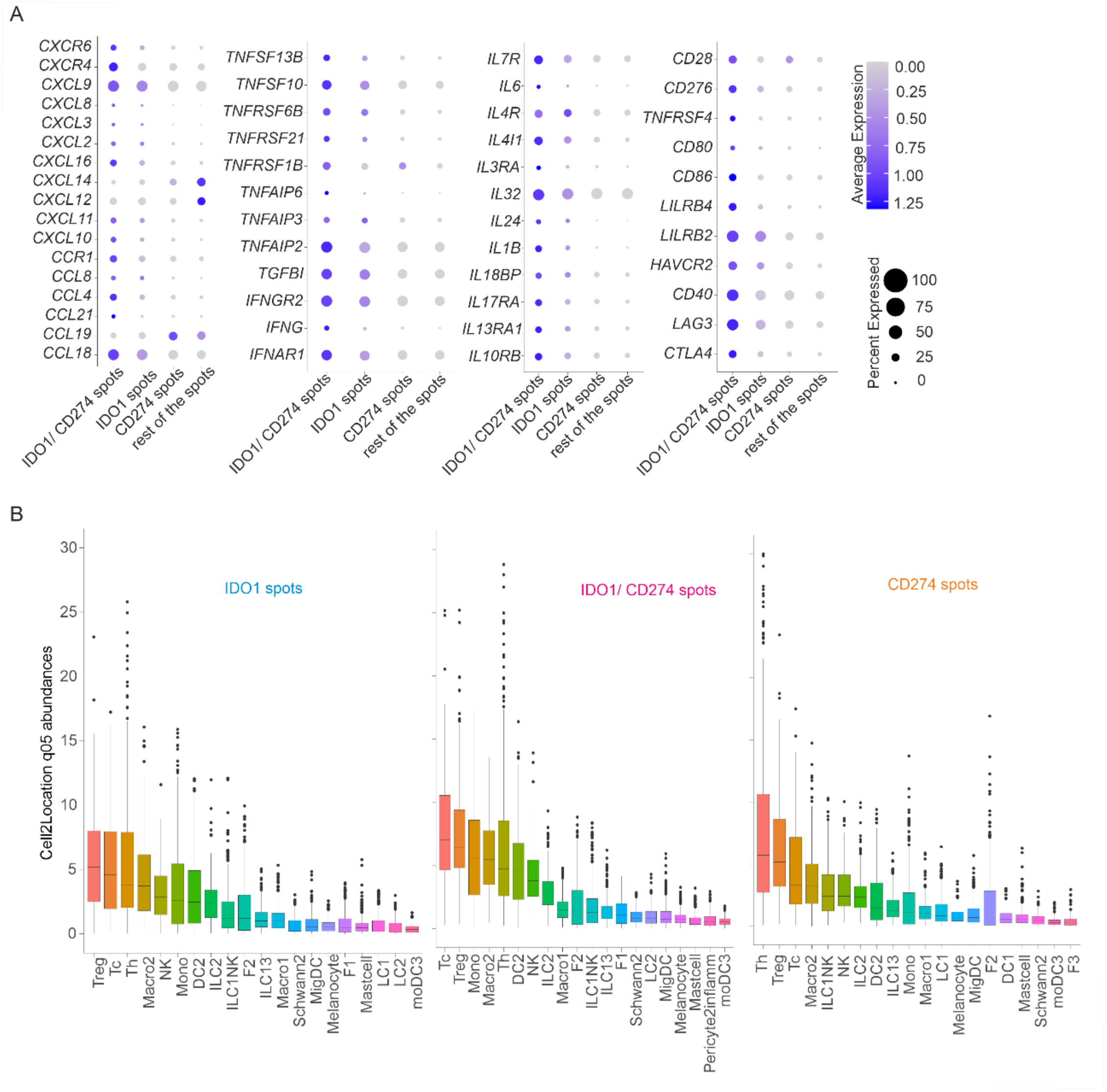

Supplemental Figure 4: DE between *IDO1*, *CD274* and *IDO1/ CD274* rich Visium spots and predicted cell types in SL_CL patients

(**A**) Differential gene expression for selected genes across IDO1, CD274 and IDO1/CD274 spot categories. Log2Fold change cut off was set at 0.3 and p value <0.01. Data shown is from all 6 patients. (**B**) Bar plot showing 5th quantile top 20 predicted cell abundances for IDO1 and CD274 classes in (A). Black line represents median value, bar indicates interquartile range.

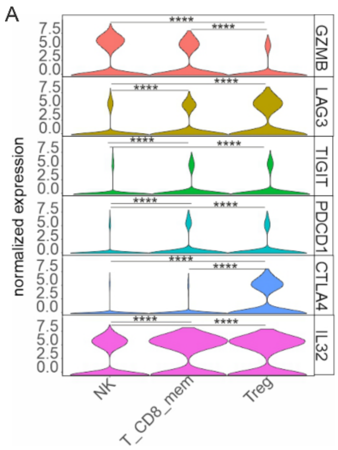

Supplemental Figure 5: CosMx expression of inhibitory receptors in IDO1, PDL1 neighbour T CD8 mem and Treg cells and all NK populations.

(**A**) Violin plot showing normalised gene expression of IL32, CTLA4, PDCD1, TIGIT, LAG3 and GZMB in NK, T CD8 memory and Treg populations. Cell IDs of T CD8 mem and Tregs were extracted from spatial network created with 4-6 neighbours of IDO1, CD274 myeloid cells and were appended to cell IDs of NK cells as a comparator. Normalised gene expression of IL32, TIGIT, CTLA4, PDCD1, LAG3 were then extracted from these cell ids and plotted in violin plot. Tukey's multiple comparisons test was used to compute significance. Data computed from n=4 SL_CL patients (Table S1)

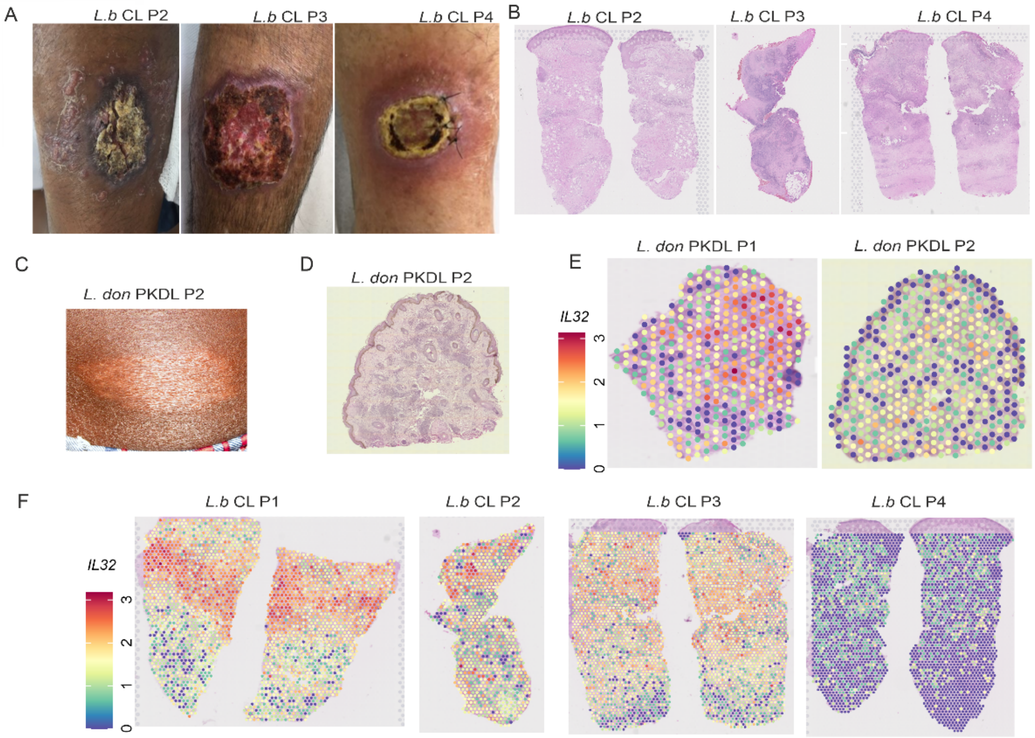

Supplemental Figure 6: Patient photographs, histology and *IL32* 10x Visium spatial maps on *L. braziliensis* and *L. donovani* infected CL and PKDL lesions respectively.

(A) CL lesions caused by *L. braziliensis.* Images for patients *L.b* CL P2-P4. (**B)** Matched H&E images of 5µm sections of lesions in (**A**). Mild inflammatory infiltrate and extensive fibrosis was seen in *L.b*. CL P2. (**C-D)** Macular PKDL lesion caused by *L. donovani.* Image for patient *L. don* PKDL P2 (**C**) and corresponding histology shown in an H&E image **(D)**. **(E-F)** Spatial plots showing gene expression for *IL32* for *L.don* PKDL_P1-P2 (**E**) and for patients *L. b* CL._P2-P4 (**F**).

Supplementary tables

Supplementary Table 1 Demographics of Sri Lankan cutaneous leishmaniasis (CL; L. donovani) patients

Patient characteristic	Summary Statistics
**Lesion & treatment duration median (range)**	
**Duration of lesion (months)**	5 (2-37)
**Amastigote grade on presentation (unit)**	2 (0-5)
**Total SSG doses needed for cure (no.)**	10 (4-29)
**Demographics, diagnostics & treatment response proportion (percentage)**	
**Age range (years)**	0-30: 3/25 (12%)<br>31-45: 9/25 (36%)<br>46-60: 10/25 (40%)<br>>60: 3/25 (12%)
**Gender**	Male: 20/25 (80%)<br>Female: 5/25 (20%)
**SSS (Slit Skin Smears) on presentation**	Positive: 18/25 (72%)<br>Negative: 7/25 (28%)
**Diagnostic PCR**	Positive: 25/25 (100%)
**Amastin (by RNA-FISH)**	Positive: 7/25 (28%)<br>Negative: 18/25 (72%)
**Treatment response**	Complete cure: 24/25 (96%)<br>Not completely healed: 1/25 (4%)

Supplementary Table 2 Demographics of Brazillian cutaneous leishmaniasis (CL; L. braziliensis) patients

Patient Characteristic	Summary Statistics
**Lesion & treatment duration median (range)**	
**Lesion size (mm²)**	1812.5 (750-5525)
**Duration of therapy (days)**	20.5 (10-120)
**Lesion duration (months)**	4.5 (2-6)
**Demographics, diagnostics & treatment route and response** ***proportion (percentage)***	
**Gender**	Male: 3 (75%)<br>Female: 1 (25%)
**Age range (years)**	31-45: 2 (50%)<br>46-60: 1 (25%)<br>>60: 1 (25%)
**Diagnostic PCR**	Positive: 4/4 (100%)
**Diagnostic Indirect immunofluorescence**	Positive: 2/4 (50%)<br>Negative: 2/4 (50%)
**Diagnostic ELISA**	Positive: 4/4 (100%)
**Treatment route**	systemic liposomal amphotericin B: 1 (25%)<br>systemic Glucantime: 2 (50%)<br>intralesional Glucantime: 1 (25%)
**Treatment score[A]**	
**Before treatment**	10: 4/4 (100%)
**1 week after treatment**	10: 4/4 (100 %)
**2 weeks after treatment**	10: 2/4 (50%)<br>8: 2/4 (50%)
**3 weeks after treatment**	10: 1/4 (25%)<br>8: 2/4 (50%)<br>5: 1/4 (25%)
**4 weeks after treatment**	9: 1/4 (25%)<br>8: 2/4 (50%)<br>3: 1/4 (25%)
**3 months after treatment**	5: 2/4 (50%)<br>0: 2/4 (50%)
**3 months after treatment**	2: 1/4 (25%)<br>0: 3/4 (75%)

[A]based on a clinician visual score where 10 is not healed and 0 is healed

Supplementary Table 3 Demographics of Indian post kala-azar leishmniasis (PKDL; L. donovani) patients

Characteristic	Patient 1	Patient 2
**Lesion history**		
**Lesion type**	Polymorphic	Macular
**History of VL**	Yes	Yes
**Year of VL**	2010	1989
**Treatment during VL**	SAG	SAG
**Year of PKDL**	2016	2013
**Lag period (years)**	6	24
**Demographics & diagnostics**		
**Age range**	0-30	31-45
**Gender**	Male	Male
**Diagnostic PCR**	Positive	Positive

Supplementary references

1. Dey NS, Senaratne S, Somaratne V, Madarasinghe NP, Seneviratne B, Forrester S, et al. Early reduction in PD-L1 expression predicts faster treatment response in human cutaneous leishmaniasis. *J Clin Invest.* 2021;131(22).
2. de Bruijn MH, and Barker DC. Diagnosis of New World leishmaniasis: specific detection of species of the Leishmania braziliensis complex by amplification of kinetoplast DNA. *Acta Trop.* 1992;52(1):45-58.
3. Moulik S, Chaudhuri SJ, Sardar B, Ghosh M, Saha B, Das NK, and Chatterjee M. Monitoring of Parasite Kinetics in Indian Post-Kala-azar Dermal Leishmaniasis. *Clin Infect Dis.* 2018;66(3):404-10.
4. Celeste BJ, Angel SO, Castro LG, Gidlund M, and Goto H. Leishmania infantum heat shock protein 83 for the serodiagnosis of tegumentary leishmaniasis. *Braz J Med Biol Res.* 2004;37(11):1591-3.
5. de Oliveira AP, de Castro MC, de Almeida AF, Souza Mde A, de Oliveira BC, Reis LC, et al. Comparison of flow cytometry and indirect immunofluorescence assay in the diagnosis and cure criterion after therapy of American tegumentary leishmaniasis by anti-live Leishmania (Viannia) braziliensis immunoglobulin G. *J Immunol Methods.* 2013;387(1-2):245-53.
6. Mao S, Zhang Y, Seelig G, and Kannan S. CellMeSH: probabilistic cell-type identification using indexed literature. *Bioinformatics.* 2022;38(5):1393-402.
7. Kleshchevnikov V, Shmatko A, Dann E, Aivazidis A, King HW, Li T, et al. Cell2location maps fine-grained cell types in spatial transcriptomics. *Nat Biotechnol.* 2022;40(5):661-71.
8. Reynolds G, Vegh P, Fletcher J, Poyner EFM, Stephenson E, Goh I, et al. Developmental cell programs are co-opted in inflammatory skin disease. *Science.* 2021;371(6527).
9. Dries R, Zhu Q, Dong R, Eng CL, Li H, Liu K, et al. Giotto: a toolbox for integrative analysis and visualization of spatial expression data. *Genome Biol.* 2021;22(1):78.
10. Hao Y, Hao S, Andersen-Nissen E, Mauck WM, 3rd, Zheng S, Butler A, et al. Integrated analysis of multimodal single-cell data. *Cell.* 2021;184(13):3573-87 e29.
11. Lex A, Gehlenborg N, Strobelt H, Vuillemot R, and Pfister H. UpSet: Visualization of Intersecting Sets. *IEEE Trans Vis Comput Graph.* 2014;20(12):1983-92.

12. Dos Santos JC, Heinhuis B, Gomes RS, Damen MS, Real F, Mortara RA, et al. Cytokines and microbicidal molecules regulated by IL-32 in THP-1-derived human macrophages infected with New World Leishmania species. *PLoS Negl Trop Dis.* 2017;11(2):e0005413.
